# More inputs of antibiotics into groundwater but less into rivers as a result of manure management in China

**DOI:** 10.1016/j.ese.2024.100513

**Published:** 2024-11-26

**Authors:** Qi Zhang, Yanan Li, Carolien Kroeze, Milou G.M. van de Schans, Jantiene Baartman, Jing Yang, Shiyang Li, Wen Xu, Mengru Wang, Lin Ma, Fusuo Zhang, Maryna Strokal

**Affiliations:** a College of Resources and Environmental Sciences, National Academy of Agriculture Green Development, Key Laboratory of Plant-Soil Interactions, Ministry of Education, China Agricultural University, 100193, China; b Earth Systems and Global Change Group, Environmental Sciences Department, Wageningen University & Research, Droevendaalsesteeg 4, Wageningen, 6708 PB, the Netherlands; c Wageningen Food Safety Research, Wageningen University and Research, Akkermaalsbos 2, 6708 WB, Wageningen, the Netherlands; d Soil Physics and Land Management Group, Wageningen University & Research, Droevendaalsesteeg 3, Wageningen, 6708 PB, the Netherlands; e Key Laboratory of Agricultural Water Resources, Hebei Key Laboratory of Soil Ecology, Center for Agricultural Resources Research, Institute of Genetic and Developmental Biology, The Chinese Academy of Sciences, Hebei, 050021, China; f Interdisciplinary Research Center for Agriculture Green Development in Yangtze River Basin, Southwest University, Tiansheng Road 02, Chongqing, 400715, China

**Keywords:** MARINA-antibiotics model, Rivers, Groundwater, Livestock production, Antibiotics

## Abstract

Antibiotics are extensively used in livestock production to prevent and treat diseases, but their environmental impact through contamination of rivers and groundwater is a growing concern. The specific antibiotics involved, their sources, and their geographic distribution remain inadequately documented, hindering effective mitigation strategies for river and groundwater pollution control caused by livestock production. Here we develope the spatially explicit MARINA-Antibiotics (China-1.0) model to estimate the flows of 24 antibiotics from seven livestock species into rivers and leaching into groundwater across 395 sub-basins in China, and examine changes between 2010 and 2020. We find that 8364 tonnes and 3436 tonnes of antibiotics entered rivers and groundwater nationwide in 2010 and 2020, respectively. Approximately 50–90% of these amounts originated from about 40% of the basin areas. Antibiotic inputs to rivers decreased by 59% from 2010 to 2020, largely due to reduced manure point sources. Conversely, antibiotic leaching into groundwater increased by 15%, primarily because of enhanced manure recycling practices. Pollution varied by antibiotic groups and livestock species: fluoroquinolones contributed approximately 55% to river pollution, mainly from pig, cattle, and chicken manure; sulfonamides accounted for over 90% of antibiotics in groundwater, predominantly from pig and sheep manure. While our findings support existing policies promoting manure recycling to mitigate river pollution in China, they highlight the need for greater attention to groundwater pollution. This aspect is essential to consider in developing and designing future reduction strategies for antibiotic pollution from livestock production.

## Highlights

• Between 90% of antibiotics found in rivers and 50% in groundwater originated from only 10%–40% of China’s basin areas.
• The total input of antibiotics into rivers decreased by 59% compared to that in ten years ago.
• Antibiotic leaching into groundwater increased by 15% from 2010 to 2020.
• Fluoroquinolones contributed to approximately 55% of river pollution in China.
• Sulfonamides accounted for over 90% of antibiotics in groundwater in China.

## Graphical abstract

**Figure:**
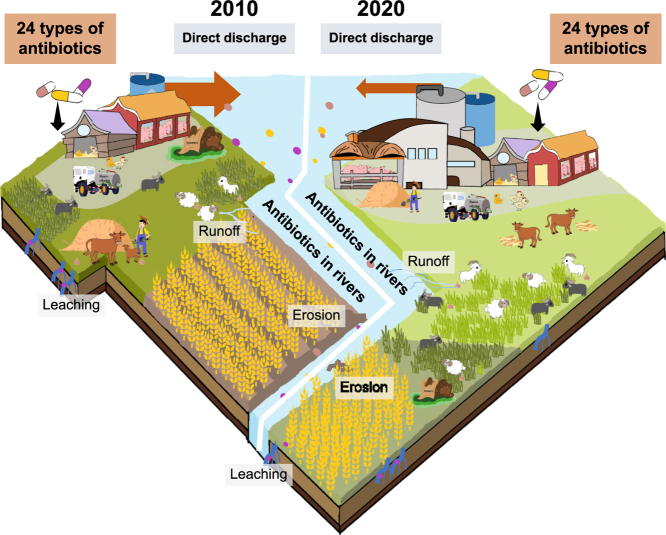

## 1 Introduction

Antibiotics are extensively used in livestock production to prevent and treat diseases [[1], [2], [3]]. In 2010, global antibiotic use in livestock production reached over 63 Gg [3,4]. Approximately 30–90% of these antibiotics are excreted in an unchanged active form during livestock excrements [1,5,6]. Annual antibiotic consumption in livestock production worldwide is projected to reach approximately 106 Gg by 2030 [4]. This may increase the use of antibiotics in the environment from livestock production.

China requires a thorough evaluation of how livestock production impacts antibiotic pollution in rivers and groundwater. China is one of the largest global users of antibiotics in livestock production globally [2]. The total use of antibiotics for livestock production was approximately 15 Gg in China in 2010 [4]. This amount is twice that of the United States [4]. This is because China had many more livestock species [7] and hardly had regulations to control antibiotic use compared to the United States [[8], [9], [10]]. Today, China still has a higher livestock population than countries such as the United States [7], which may lead to more antibiotic use. Manure management differs from the past. Historically, livestock manure has been used as an organic fertilizer, especially in crop production [11]. Since the 1990s, large amounts of manure were not used in crop production [9,12]. Synthetic fertilizers largely replaced manure. As a result, considerable amounts of manure were dumped into surface waters. Since the 2000s, the national government has introduced various environmental policies to avoid water pollution by directly discharging manure through more manure recycling on the land [13,14]. Farmers start using manure more often as an organic fertilizer.

Because of the incomplete absorption and partial metabolism, unchanged active forms of antibiotics are excreted from the bodies of livestock species in the manure [1,5,6]. Some amounts of antibiotics in manure can directly enter rivers through direct discharges of manure [[8], [9], [10]]. In contrast, some antibiotics can enter agricultural land through manure application. From fertilized land, antibiotics can enter rivers through runoff and soil erosion and leach through deeper soil layers into groundwater [2,10]. Antibiotics have been widely detected in soils and waters in China [2,3]. Antibiotics in the environment can disturb biological and microbial communities and promote the transition and spread of antibiotic-resistant genes [15].

A spatially explicit assessment of antibiotic inputs to rivers and groundwater from livestock does not exist for Chinese sub-basins. Meanwhile, our understanding of the contribution of different livestock species to antibiotic-related water pollution in rivers and groundwater remains limited. Previous studies have focused on specific sources, locations, antibiotics, and water systems [3,5,16,17]. Many studies have reported the concentration of antibiotics in livestock farms, wastewater, or manure [18,19] and have covered specific livestock farms or species [18,20,21]. These studies provided important insights into local water pollution with antibiotics to better understand the local contributions of antibiotics and livestock species [3,5,17,18,20,21]. Local stakeholders and decision-makers can use this information to develop policies and regulations. However, such insights are hardly available to cover the whole of China.

Several models have been used to quantify antibiotics-related water pollution in China [1,16,17]. For instance, a Level-III fugacity model is applied to China [1]. This model allows for the spatially explicit analysis of 58 basins of China, which enables quantifying the contribution of livestock species to rivers in 2013 [1]. This model does not quantify antibiotic leaching into groundwater or consider the diffusion of antibiotic inputs to rivers in 2020. The model only considers the antibiotic usage by pigs, chickens, and others (total weight of meat of sheep, cows, and fish) in China [1]. River sub-basins are crucial for better understanding spatial variability in pollution levels, particularly in large basins like the Yellow and Yangtze Rivers. Existing models hardly focus on sub-basin scales for the whole of China. An exception is the family of the MARINA models (Model to Assess River Inputs of pollutaNts to seAs). These models run at the sub-basin scale for nutrients, plastics, and chemicals [10,[22], [23], [24], [25]]. The models have been widely applied globally, nationally (China and Europe), and in individual lakes [10,[26], [27], [28], [29], [30], [31]]. However, such models do not exist for antibiotics.

Many antibiotics are used in Chinese livestock production. Among them, 24 antibiotics are extensively used in livestock production and frequently detected in China [1,32]. These 24 antibiotics are grouped into six categories, i.e., Sulfonamides, Tetracyclines, Fluoroquinolones, Macrolides, β-lactams, and Lincosamides (Supplementary Materials Fig. S1). Antibiotics used for livestock production accounted for 52% of the total antibiotics in China in 2013 [1]. Zhang et al. [1] reported that these 24 antibiotics contributed to over 55% of the total usage of antibiotics in China in 2013 based on survey data.

Thus, there is a lack of quantitative information at the sub-basin scale on the effects of livestock distribution and manure management on antibiotic water pollution in China. In recent decades, the Chinese government has exhibited increased proactivity and assertiveness in addressing diffuse source pollution caused by agriculture. Agricultural Green Development (AGD) was proposed as a national strategy for sustainable development in China at the 19th National People’s Congress in 2017 [33]. AGD aims to increase environmental quality while satisfying food demand to achieve sustainable agriculture. However, to the best of our knowledge, we still lack knowledge on simultaneously assessing livestock production relocation, improving manure management, and the resulting impacts on antibiotic water pollution during the period of 2010–2020. The period from 2010 to 2020 is important when looking at the history of agricultural policies in China. Before 2010, agricultural policies for manure management were generally limited in China [8,9,12]. From 2010 until 2020, various agricultural policies have been introduced to facilitate more manure recycling on land to avoid direct discharges of manure to rivers and, thus, reduce river pollution [[33], [34], [35]]. Some policies aim to shift livestock production from the south to the north [[35], [36], [37]]. Examples are the ‘14th Five-Year National Agricultural Green Development Plan’ [33], ‘Livestock and Poultry Manure Utilization Action Plan (2017–2020)’ [34], and China’s livestock relocation policies [36]. These policies may influence water pollution with antibiotics in China. However, the period of 2010–2020 has not been well studied for water pollution with antibiotics but needed to support the formulation of water pollution control strategies to achieve AGD policies in China [38] as well as to support the United Nations' Sustainable Development Goals 6 (clean water) and 12 (sustainable food production) [39]. Sub-basin analyses may help better understand pollution hotspots and their sources to formulate sustainable AGD solutions for clean water in China and elsewhere.

Our main research objective is to estimate the flow of 24 antibiotics from seven livestock species into rivers and leaching into groundwater in 395 sub-basins in China and to examine changes in antibiotic water pollution between 2010 and 2020. The spatially explicit MARINA-Antibiotics (China-1.0) model (Model to Assess River Inputs of pollutaNts to seAs for Antibiotics in freshwater) is developed and evaluated for this study. Our model results can be used to prioritize sub-basins, livestock species, and antibiotic groups in water pollution control. Other countries that experience antibiotic pollution from intensive livestock production can use our model as a tool to better understand which antibiotic group (e.g., Sulfonamides), where (sub-basins), and from which livestock species (e.g., cattle) enter rivers and groundwater. This could raise the attention of the public, policymakers, and other stakeholders on the need to consider antibiotics in national water quality policies and monitoring programs. This study will contribute to developing AGD strategies to reduce antibiotic use in Chinese waters and elsewhere.

## 2 Materials and methods

### 2.1 MARINA-antibiotics model

**Fig. 1:**
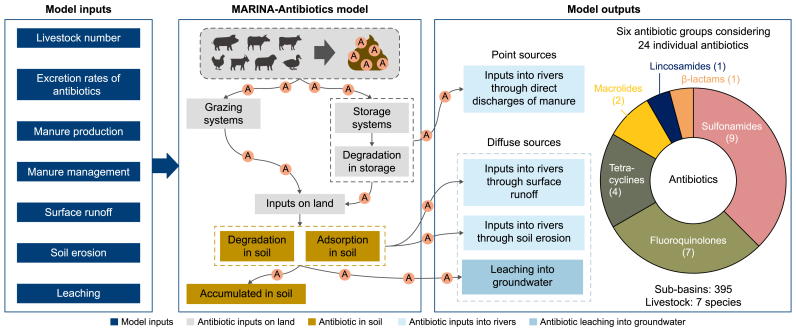
Overview of the MARINA-Antibiotics (China-1.0) model, focusing on livestock manure. MARINA-Antibiotics is short for Model to Assess River Inputs of pollutaNts to seAs for Antibiotics in freshwater. The model considers seven livestock species: pigs, buffaloes, cattle, chickens, goats, sheep, and ducks. ‘A’ denotes antibiotics. Twenty-four antibiotics from six groups are considered in this model: Sulfonamides, Tetracyclines, Fluoroquinolones, Macrolides, β-lactams, and Lincosamides. For details on model inputs, see Tables S1–S4 in the Supplementary Materials. Sources: the MARINA-Antibiotics (China-1.0) model (Section 2.1).

MARINA-Antibiotics is short for Model to Assess River Inputs of pollutaNts to seAs for Antibiotics in freshwater. We developed the first version for antibiotic loadings into rivers and groundwater (via leaching) from livestock manure (Fig. 1) and applied it to 395 Chinese sub-basins. Our MARINA-Antibiotics (China-1.0) model was based on an existing sub-basin-scale MARINA-Multi (Global-2.0) modeling approach [10] and integrated chemical approaches [40,41]. Our model quantified antibiotics in rivers and groundwater using consistent model inputs (e.g., livestock density) in space (e.g., sub-basins) and time (e.g., annual). Our selection of the years 2010 and 2020 was justified for two primary reasons: (1) the implementation of key Chinese agricultural policies during this period [[33], [34], [35], [36], [37],^42^] and (2) the availability of data for these years (see details in Supplementary Materials). Thus, we applied the model to 2010 and 2020 to demonstrate the potential effect of the implemented national agricultural policies on water pollution with antibiotics in China.

The MARINA-Antibiotics (China-1.0) model used a lumped approach to quantify annual antibiotic inputs to rivers and leaching to groundwater from seven livestock species. The livestock species included pigs, buffaloes, cattle, chickens, goats, sheep, and ducks. In our study, we selected 24 antibiotics from six main categories (Supplementary Materials Fig. S1):

(1) Sulfonamides (sulfaquinoxaline, sulfathiazole, sulfamethoxazole, sulfamethazine, sulfameter, sulfamonomethoxine, sulfaguanidine, sulfadiazine, and sulfachlorpyridazine);
(2) Tetracyclines (oxytetracycline, doxycycline, tetracycline, and chlortetracycline);
(3) Fluoroquinolones (pefloxacin, ofloxacin, norfloxacin, fleroxacin, enrofloxacin, difloxacin, and ciprofloxacin);
(4) Macrolides (tylosin and roxithromycin);
(5) β-lactams (penicillin-G);
(6) Lincosamides (lincomycin).

We considered the direct discharge of manure to rivers, degradation (persistence) of antibiotics in manure during storage, soil degradation and adsorption of antibiotics, runoff, soil erosion, and leaching to determine the fate and transfer of antibiotics from livestock production to rivers and groundwater (Fig. 1, equations (1), (2), (3), (4), (5), (6), (7), (8), (9)). The input data were processed using ArcGIS, and many model parameters differ among sub-basins (see Tables S1 and S9 in the Supplementary Materials for details).

Below, we presented calculations for the inputs of antibiotics to rivers and groundwater from livestock production. In the MARINA-Antibiotics (China-1.0) model, the top 60 cm of the soil was regarded as the root zone. The 60–200 cm region of the soil was considered the last soil layer before antibiotic leaching into groundwater, following the definition from Poggio et al. [43] and Arrouays et al. [44]. In the top 60 cm of the soil, antibiotics in soil particles and solutions could be transported to rivers via soil erosion and runoff. Below 200 cm of the soil, we estimated the antibiotics that remain in the soil solution after runoff losses, and these antibiotics could further leach into groundwater. The depth of 200 cm was chosen based on its relevance to the scope of the study. This depth captured both relevant processes in the soil profile and considered the potential vertical movement of antibiotics in deeper soil layers. In existing studies [[44], [45], [46], [47], [48]], 200 cm was used as a common depth for examining soil properties, leaching patterns, and transport of pollutants. For example, Arrouays et al. [44] indicated that 200 cm is pragmatic for soil sampling, providing reliable observations of soil properties, even in thick soil. For assessing pollutants leaching into groundwater, Zhang et al. [45] explored the impacts of agricultural fertilization on nitrate in soil and leaching into groundwater, including the movement of agricultural nitrate to a depth of 200 cm. This depth also typically included the root zone of most crops (e.g., corn, wheat, alfalfa, and soybean) and reached the subsoil [46]. Contaminants moving to this depth may potentially entered groundwater or deeper soils.

#### 2.1.1 Modeling inputs of antibiotics into rivers

The model distinguished between diffuse and point sources of river pollution. The annual inputs of antibiotics into the rivers from point and diffuse sources were calculated using equation (1). Point sources of antibiotics in rivers resulted from the direct discharges of livestock manure from storage systems (equation (2)). Diffuse sources of antibiotics in rivers resulted from runoff and soil erosion after the application of manure to agricultural land, which is corrected for degradation processes in the soil. Antibiotic inputs to rivers from diffuse sources were calculated as a function of antibiotic inputs to agricultural land from storage and grazing systems, soil retention, erosion, and runoff (equations (3), (4), (5), (6), (7), (8)).

(1) $${RS}_{A,i,j}={RSdif}_{A,i,j}+{RSpnt}_{A,i,j}$$

(2) $${RSpnt}_{A,i,j}=S_{ss,A,i,j}\times {fr}_{d,i,j}$$

(3) $${RSdif}_{A,i,j}={RSsr}_{A,i,j}+{RSes}_{A,i,j}$$

(4) $${RSsr}_{A,i,j}={Ssol}_{A,i,j}\times {FEsr}_{j}$$

(5) $${RSes}_{A,i,j}={Spar}_{A,i,j}\times {FEes}_{j}$$

In these equations,

${RS}_{A,i,j}$ is the total antibiotic (*A*) input to rivers (*RS*) from the manure of livestock species (*i*) in the sub-basin (*j*) (kg antibiotics per year).

${RSdif}_{A,i,j}$ is the antibiotic (*A*) input to rivers (*RS*) from the manure of livestock species (*i*) resulting from diffuse sources (*dif*) in the sub-basin (*j*) (kg antibiotics per year).

${RSpnt}_{A,i,j}$ is the antibiotic (*A*) input to rivers (*RS*) from the manure of livestock species (*i*) resulting from point sources (*pnt*) in the sub-basin (*j*) (kg antibiotics per year).

$S_{ss,A,i,j}$ is the excretion of the antibiotic (*A*) in the manure of livestock species (*i*) in the storage system (*ss*) in the sub-basin (*j*) (kg antibiotics per year). This parameter was calculated according to the equations in Table S1 of the Supplementary Materials. See more details in Tables S1–S4 of the Supplementary Materials.

${fr}_{d,i,j}$ is the fraction (*fr*) of direct discharges (*d*) of manure to rivers from livestock species (*i*) in the sub-basin (*j*) (0–1). This fraction differs among livestock species.

${RSsr}_{A,i,j}$ is the antibiotic (*A*) inputs to rivers (*RS*) from the manure of livestock species (*i*) resulting from surface runoff (*sr*) in the sub-basin (*j*) (kg antibiotics per year).

${RSes}_{A,i,j}$ is the antibiotic (*A*) input to rivers (*RS*) from the manure of livestock species (*i*) resulting from soil erosion (*es*) in the sub-basin (*j*) (kg antibiotics per year).

${Ssol}_{A,i,j}$ is the amount of antibiotic (*A*) from the manure of livestock species (*i*) available in the soil solution (*Ssol*) after adsorption and degradation in the sub-basin (*j*) (kg antibiotics per year).

${FEsr}_{j}$ is the export fraction (*FE*) of antibiotics that entered rivers in the soil solution resulting from surface runoff (*sr*) in the sub-basin (*j*) (0–1). This export fraction was calculated as a function of surface runoff and precipitation different per sub-basin. Details on calculations are in Tables S1–S4 of the Supplementary Materials.

${Spar}_{A,i,j}$ is the total amount of antibiotic (*A*) from the manure of livestock species (*i*) available in the soil particles (*Spar*) after adsorption and degradation in the sub-basin (*j*) (kg antibiotics per year).

${FEes}_{j}$ is the export fraction (*FE*) of antibiotics that enter rivers in the soil particles resulting from soil erosion (*es*) in the sub-basin (*j*) (0–1). This export fraction varied among sub-basins. More details are in Tables S1–S4 of the Supplementary Materials.

Antibiotics in soil solution and soil particles after adsorption and degradation were calculated as a function of various processes (equations (6), (7)) as follows:

(6) $${Spar}_{A,i,j}={WSdif}_{A,i,j}\times {FS}_{par,A,j}\times {FS}_{de,A,j}$$

(7) $${Ssol}_{A,i,j}={WSdif}_{A,i,j}\times {FS}_{sol,A,j}\times {FS}_{de,A,j}$$

where,

${WSdif}_{A,i,j}$ is the application of antibiotic (*A*) from the manure of livestock species (*i*) in the sub-basin (*j*) to agricultural land (kg antibiotics per year).

${FS}_{par,A,j}$ and ${\;FS}_{sol,A,j}$ are the adsorption fractions (*FS*) of antibiotic (*A*) in the soil particle (*par*) and solution (*sol*) in the sub-basin (*j*), respectively (0–1). The adsorption fractions of antibiotics were calculated as a function of the linear adsorption constant (K_d_ value) of antibiotics (L kg^−1^) and the maximum water-holding capacity of soil based on the soil textures. The adsorption fractions of antibiotics in the soil particle and solution varied among sub-basins.

${FS}_{de,A,j}$ is the degradation (*de*) fraction (*FS*) of antibiotic (*A*) in the soil after manure application from the manure management systems. This fraction was calculated as a function of the degradation rate of antibiotics in the soil ($k_{j}$) and the degradation time duration in the soil ($t_{j}$). This degradation fraction differed among sub-basins. More details are in Tables S1–S4 of the Supplementary Materials.

Adsorption processes reflect the amount of antibiotics attached to soil particles and in solution. Physical (e.g., the maximum water-holding capacity of soil-related antibiotic adsorption) and chemical (e.g., Kd value, the linear adsorption constant) processes influence the adsorption of antibiotics in the soil. The maximum water-holding capacity of the soil depends on soil texture. Here, we distinguished the dominant soil textures among sub-basins based on the data from the National Earth System Science Data Center (NESSDC) [49]. According to Pan and Chu [50] and Geohring et al. [51], we considered the dominant maximum water-holding capacity based on soil textures among sub-basins. The degradation of antibiotics in the soil is influenced by physical (such as soil temperature and soil saturated water content, which relates to antibiotics degradation), chemical (such as soil pH, soil organic carbon content, and the half-life of antibiotics-related degradation), and biological (such as biological responses to soil changes associated with antibiotic degradation) processes. These processes were incorporated into our model according to the approach of Tang and Maggi [41] and Wöhler et al. [40] (details are in Tables S1–S4 of the Supplementary Materials). Each sub-basin has different model inputs to represent the physical, chemical, and biological processes in the soil for the degradation of antibiotics.

Antibiotic inputs to agricultural land from grazing and storage systems were calculated using equation (8). Livestock manure was considered an organic fertilizer for agricultural land. In grazing systems, manure-containing antibiotics entered the land directly. For storage systems, the input of antibiotics to agricultural land was calculated as a function of manure production, livestock number, and the degradation (persistence) rates of antibiotics during the manure management practices (e.g., storage, composting, and anaerobic digestion) according to the livestock species and antibiotics. This implied that manure was collected during storage before being applied to agricultural land and was also corrected for direct discharges of manure to rivers. Antibiotic losses during storage were associated with, for example, the degradation of antibiotics during manure management practices in storage systems.

(8) $${WSdif}_{A,i,j}={WSdif}_{A,sg,i,j}+{WSdif}_{A,ss,i,j}$$

In this equation,

${WSdif}_{A,sg,i,j}$ is the application of antibiotic (*A*) to agricultural land from the manure of livestock species (*i*) from grazing system (*sg*) in sub-basin (*j*) (kg antibiotics per year). This parameter was calculated according to the equations in Table S1 of the Supplementary Materials. See more details in Tables S1–S4 of the Supplementary Materials.

${WSdif}_{A,ss,i,j}$ is the application of antibiotic (*A*) to agricultural land from the manure of livestock (*i*) from the storage system (*ss*) after correcting for the direct discharges of manure to rivers and manure management practices (e.g., storage, composting, and anaerobic digestion) in the sub-basin (*j*) (kg antibiotics per year). The model input was calculated using the following equation: ${WSdif}_{A,ss,i,j}=S_{ss,A,i,j}\times \left(1-{fr}_{d,i,j}\right)$. See more details in Supplementary Materials Tables S1–S4.

#### 2.1.2 Modeling leaching of antibiotics to groundwater

We listed the main equation that was used to calculate the amount of antibiotics leaching into groundwater from manure application on agricultural land (the amount of antibiotics leaving the soil layer below 200 cm):

(9) $${GW}_{A,i,j}=\left({Ssol}_{A,i,j}-{RSsr}_{A,i,j}\right)\times {fr}_{le,A,j}$$

where,

${GW}_{A,i,j}$ is the total input of antibiotic (*A*) leaching into groundwater (*GW*) from the manure application on agricultural land from livestock species (*i*) in the sub-basin (*j*) (kg antibiotics per year).

${fr}_{le,A,j}$ is the leaching fraction (${fr}_{le}$) of the antibiotic (*A*) from the soil solution below 200 cm of soil in the sub-basin (*j*) (0–1). This fraction was calculated as a function of the soil texture, the antibiotics available in the soil solution, and the maximum water-holding capacity of the soil (details in Tables S1–S4 of the Supplementary Materials). This model input differed among sub-basins.

#### 2.1.3 Model inputs

Livestock numbers in 2010 were derived from Li et al. [10]. We calculated the trends of the livestock number at the provincial level from 2010 [52] and 2020 [53] based on Chinese Statistic Yearbooks. The livestock numbers and their spatial distribution for 2010 and 2020 were derived using the following steps based on the approaches of Li et al. [24] and Zhang et al. [54]. We assigned the provincial changes in livestock numbers between 2010 and 2020 to corresponding 0.5-degree grids. Then, we multiplied the changes by the livestock number at 0.5-degree grids in 2010. We summed the values over the grids for the corresponding sub-basins to obtain the number of livestock with changed spatial distributions in 2020 per sub-basin. Livestock numbers differed among livestock species and sub-basins. See more details in Supplementary Materials Fig. S2 and Tables S1–S4, and S9.

The fractions of direct discharges of livestock manure into rivers by livestock species in 2010 were derived from Li et al. [10]. Due to data availability, we updated these fractions by livestock species in 2020 based on trends in manure not recycled to agricultural land derived at the provincial level by Zhu et al. [55] to obtain input data at the sub-basin scale. We aggregated the provincial data to the sub-basin scale following the approaches of [10,54,56]. The excretion rates of 24 antibiotics by livestock species were calculated based on previous studies (Supplementary Materials Table S3) [1,[18], [19], [20], [21],^57^]. This fraction differed among livestock species. See more details in Supplementary Materials Fig. S2 and Tables S1–S4, and S9.

The export fraction of antibiotics that enter rivers in the soil solution due to surface runoff in the sub-basin in 2010 was derived from Li et al. [24]. The Variable Infiltration Capacity hydrological model provided data for precipitation and natural river discharges for the period up to 2020 [58]. Then, we calculated the average annual precipitation per sub-basin by statistically averaging 30-year annual runoff per sub-basin. This was calculated based on the 30-year average annual natural river discharge in the sub-basin divided by their drainage area. Finally, we calculated the export fraction in 2020 based on the 30-year (1990–2020) averaged runoff divided by the 30-year (1990–2020) averaged precipitation per sub-basin following the approach of Li et al. [24]. See more details in Supplementary Materials Fig. S2 and Tables S1–S4, and S9. This model parameter was different per sub-basin. This export fraction reflected land use change and pollutants transported from land to rivers through surface runoff. Sub-basins with higher export fractions received more pollutants entering their rivers through surface runoff than those with lower export fractions.

### 2.2 Model evaluation approach

We evaluated our model following the ‘building trust’ approach of Strokal et al. [59], which was developed for large-scale water quality models, and validation is challenging [24,60]. We selected four options to build trust in the model approach, inputs, and outputs. Option 1 compared our model inputs with existing datasets. Option 2 compared our model outputs with those of existing studies. Here, we collected available data, including those from modeling studies and observations. Option 3 compared the spatial variability of pollution hotspots with existing studies. Option 4 used expert knowledge to verify uncertain model parameters for which data are limited in space and time. The results of these four options are presented in the Results and Discussion section.

### 2.3 Definition of pollution hotspots

We defined ‘pollution hotspots’ for the antibiotic input into rivers and groundwater following the approach of Li et al. [10]. In descending order, we ranked sub-basins based on the inputs of antibiotics per km^2^ of the sub-basin areas. Consequently, we have inputs ranging from Level I (lower inputs) to Level V (higher inputs). For rivers, the input of antibiotics ranged from 0 to 0.2 g km^−2^ year^−1^ (Level I), 0.2–2 g km^−2^ year^−1^ (Level II), 2–32 g km^−2^ year^−1^ (Level III), 32 to 700 (Level IV), and 700 to 4468 (Level V). For groundwater, antibiotic inputs ranged from 0 to 0.1 g km^−2^ year^−1^ (Level I), 0.1–0.2 g km^−2^ year^−1^ (Level II), 0.2–2 g km^−2^ year^−1^ (Level III), 2–3 g km^−2^ year^−1^ (Level IV), and 3–31 g km^−2^ year^−1^ (Level V). The top 25% of sub-basins were considered pollution hotspots, for which inputs of antibiotics in rivers and groundwater fall under Levels IV and V.

## 3 Results and Discussion

### 3.1 Antibiotic river pollution

**Fig. 2:**
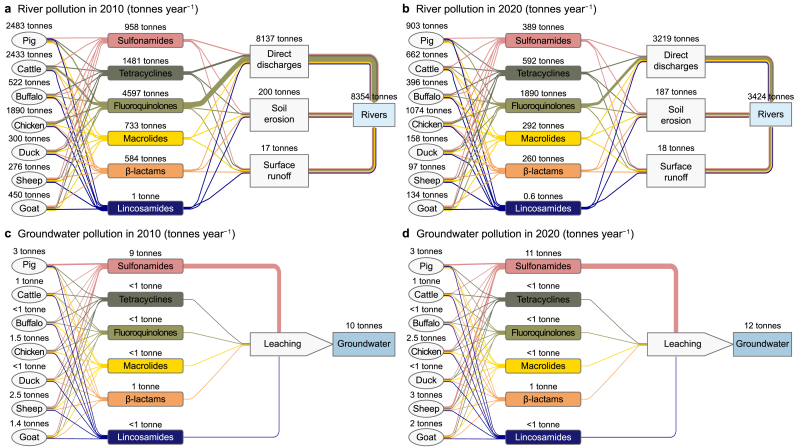
Annual flows of antibiotics from livestock manure to rivers (**a**, **b**) and groundwater (**c**, **d**) in China in 2010 (**a**, **c**) and 2020 (**b**, **d**) (tonnes of antibiotics per year). Sources: the MARINA-Antibiotics (China-1.0) model (see Section 2.1 for the model description).

We modeled that approximately 8354 and 3424 tonnes of all 24 antibiotics entered Chinese rivers from livestock production in 2010 and 2020, respectively (Fig. 2a and b). This implies that the total input of antibiotics into rivers decreased by approximately 59% from 2010 to 2020 for China as a whole. This was largely due to decreased direct manure discharges into rivers (fewer manure point sources). In 2010, the contribution of direct manure discharges to the total input of antibiotics in all rivers in China was 8137 tonnes. This decreased to 3219 tonnes in 2020 (Supplementary Materials Tables S6–S9). The ‘14th Five-Year National Agricultural Green Development Plan’ has called for an increase in the use of livestock manure on land to 76% in 2020 in China as a whole [33,34]. This could be attributed to the fact that more manure was recycled on land to avoid its direct discharge into rivers, which is the potential effect of the introduced agricultural policies in 2020 compared to 2010. Our results showed that fluoroquinolones accounted for 55% and 56% of rivers' antibiotics in 2010 and 2020, respectively (Fig. 2). In 2010, pig and cattle manure were the dominant contributors to antibiotic pollution in rivers. In 2020, antibiotic inputs to rivers were mainly pig and chicken manure.

River pollution varied largely among the 395 Chinese sub-basins (Supplementary Materials Fig. S18). We distinguished between five pollution levels (see Section 2.3 for the definition). In 2010, river sub-basins in Levels I–II received less than 2 g of antibiotics per km^2^ of sub-basin area annually (Supplementary Materials Fig. S18). This resulted in approximately 3 tonnes of all selected antibiotics entering rivers in these sub-basins. The contribution of runoff in this river pollution was 45%, and the contribution of soil erosion was 55% (diffuse sources). Most of the Level I–II sub-basins were in the western region of China, covered 38% of the total surface area, and accommodated 2% of the total human population in China in 2010 (Supplementary Materials Figs. S6 and S7). Between 2010 and 2020, the total antibiotic input to all rivers in these sub-basins increased by 18%. However, changes in antibiotic pollution during 2010–2020 ranged from −49% (decrease) to +270% (increase) among sub-basins of Levels I–II (Fig. S19). This implies that rivers in some western sub-basins became cleaner (decreased pollution), whereas rivers in other sub-basins in the north and northwest became more polluted (increased pollution) during 2010–2020. The livestock numbers of northern sub-basins in Levels I–II increased by 3%–226% between 2010 and 2020 among sub-basins (Supplementary Materials Figs. S3–S5). Moreover, the application of antibiotics to livestock manure on agricultural land increased by 33% between 2010 and 2020 (Supplementary Materials Table S9). The combined effects of the changed spatial distribution of livestock production and the application of manure resulted in the northern rivers in Level I–II sub-basins receiving more antibiotics by 2020 (Supplementary Materials Fig. S19). Fluoroquinolone and Sulfonamide groups were more responsible for river antibiotic pollution in these sub-basins in 2020 than in 2010 (Fig. 4). They mainly originated from cattle and sheep manure (Fig. 4; Supplementary Materials Fig. S8).

**Fig. 3:**
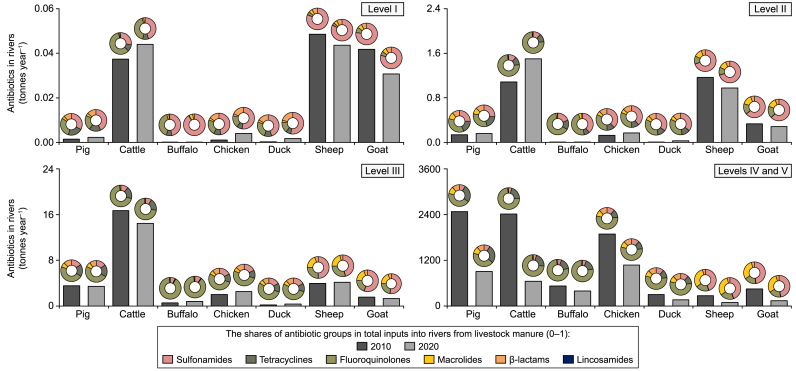
River pollution by antibiotics in 395 Chinese sub-basins according to pollution levels and by livestock species in 2010 and 2020. The bar graphs show antibiotic inputs from livestock manure (tonnes year^−1^) to rivers. The pie charts show the share of antibiotic groups in the total antibiotic inputs to rivers from livestock manure (0–1). Levels I–V refer to the pollution levels of total river antibiotic inputs (see Section 2.3 for the definition). Fig. S19 in the Supplementary Materials shows the changes in antibiotic inputs into rivers (%) between 2010 and 2020. Sources: the MARINA-Antibiotics (China-1.0) model (see Section 2.1 for the model description).

**Fig. 4:**
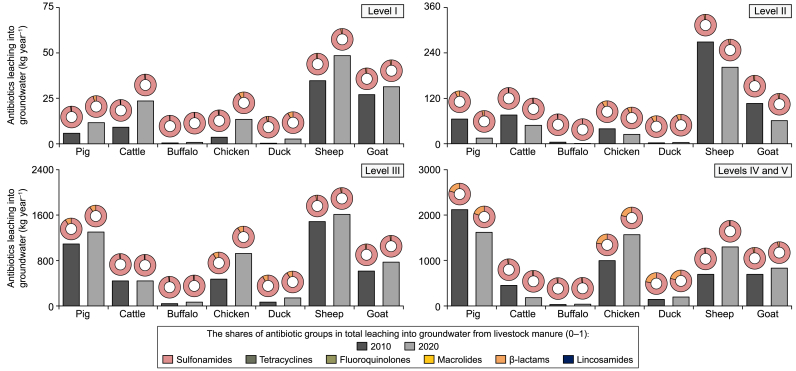
Antibiotics leaching into groundwater in 395 Chinese sub-basins according to pollution level and by livestock species in 2010 and 2020. The bar graphs show antibiotic leaching from livestock manure (kg year^−1^) into groundwater. The pie charts show the share of antibiotic groups in the total leaching into groundwater from livestock manure (0–1). Levels I–V refer to the pollution levels of the total antibiotic leaching into groundwater (definition see Section 2.3). Fig. S20 in the Supplementary Materials shows the changes in antibiotic leaching into groundwater (%) between 2010 and 2020. Sources: the MARINA-Antibiotics (China-1.0) model (see Section 2.1 for the model description).

Rivers in Level III sub-basins received 31 tonnes of antibiotics in 2010, mainly from diffuse sources in which the share of soil erosion was considerable (82% of total antibiotics in rivers, Supplementary Materials Fig. S18). Most of the Level III sub-basins were in northeastern and southwestern China. These sub-basins covered 22 % of the total surface area. They accommodated 13 % of the total population in China in 2010 (Supplementary Materials Figs. S6–S7). Between 2010 and 2020, the total antibiotic inputs to all rivers in these sub-basins decreased by 11%. As a result, 27 tonnes of antibiotics from livestock production entered rivers in the Level III sub-basins in 2020. Most northeastern Level III sub-basins experienced increased antibiotic pollution in rivers (over 50% increase, Supplementary Materials Fig. S19). Most southern sub-basins of Level III were estimated to have decreased by more than 25% in total antibiotic river pollution by the year 2020 (Supplementary Materials Fig. S19). These changes could be attributed to the greater increases in livestock numbers and manure recycling in the northern sub-basins than in the southern sub-basins in Level III by 2020 (Supplementary Materials Figs. S3–S5 and Table S9). In addition, the Level III sub-basins generally had moderate surface runoff and soil erosion, which could facilitate the antibiotics transport through the topsoil layer into rivers (Supplementary Materials Fig. S19). Fluoroquinolones were the predominant antibiotic group, accounting for 58% of the total antibiotic input to river sub-basins in Level III for the years 2010 and 2020 (Fig. 4; Supplementary Materials Fig. S9). Cattle manure was the main source of Fluoroquinolones and Tetracyclines in rivers of these sub-basins in both 2010 and 2020 (Fig. 4; Supplementary Materials Fig. S8).

Over 90% of antibiotics in the rivers originated from 40% of the basin area in China (Supplementary Materials Figs. S7 and S18). These sub-basins were identified as pollution hotspots (Levels IV and V) in this study (Supplementary Materials Fig. S18). Most of this amount was from point sources that accounted for over 94% of total antibiotics in rivers of these sub-basins in 2010 and 2020. In 2010 and 2020, rivers in hotspot sub-basins received 8321 and 3393 tonnes of total antibiotic inputs, respectively. These sub-basins were in central and southern China (Supplementary Materials Fig. S18), which accommodated approximately 84% of the total population in 2020 (Supplementary Materials Fig. S6). Between 2010 and 2020, the antibiotic inputs to rivers of hotspot sub-basins decreased by 59%. However, the changes in antibiotic inputs into rivers ranged from −75% (decrease) to +62% (increase) among Levels IV–V sub-basins (Supplementary Materials Fig. S19) during this period. For hotspot sub-basins located in central and southeastern China, our model estimated a decrease of over 50% in river pollution during 2010–2020 (Supplementary Materials Fig. S19). Rivers in some southwestern hotspot sub-basins also became cleaner, with a decrease of more than 25% in total antibiotic inputs by 2020 compared to 2010 (Supplementary Materials Fig. S19). Total antibiotic inputs to rivers in a few hotspot sub-basins in southwestern China increased by 0–25% between 2010 and 2020 (Supplementary Materials Fig. S19). This may also be relevant to the implication of agricultural policies for forbidden direct discharges of livestock manure to rivers and the changed spatial distribution of livestock production during 2010 and 2020. Higher pollution levels in hotspot sub-basins may also largely be associated with higher surface runoff and soil erosion compared to those in the other sub-basins (Supplementary Materials Table S6 and Fig. S18). The proportion of antibiotic groups in rivers varied considerably among sub-basins (Supplementary Materials Fig. S9). Fluoroquinolones contributed more than 40% of the total antibiotic inputs to rivers in hotspot sub-basins between 2010 and 2020 (Supplementary Materials Fig. S9). Pig and cattle manure were the dominant contributors to river pollution with antibiotics from livestock manure in 2010 (Fig. 4; Supplementary Materials Fig. S6). Compared to 2010, the contribution of chicken manure to total river pollution in hotspot sub-basins increased by 46% in 2020 (Fig. 4). Hotspot sub-basins received approximately 2400 tonnes of antibiotic inputs to rivers from pig and chicken production in 2020.

### 3.2 Antibiotic groundwater pollution

Our model estimated that 10 and 12 tonnes of antibiotics were leached into groundwater nationally in 2010 and 2020, respectively (Fig. 2c and d). This implies that the total leaching of antibiotics into groundwater increased by 15% during 2010–2020 for China as a whole. This could be a result of more recycled manure on land and changed spatial distribution of livestock species in 2020 compared to that in 2010 (Supplementary Materials Figs. S3–S5 and Table S9). For example, in 2010, 1587 tonnes of antibiotics in manure were applied on land in northern sub-basins of China. This amount increased to 2183 tonnes in 2020 (Supplementary Materials Table S9). Increased manure recycling was facilitated by existing agricultural policies to avoid direct manure discharges from 2010 to 2020 [33,34]. Our results showed that Sulfonamides contributed to over 90% of the total antibiotic leaching into groundwater during 2010–2020 (Fig. 2c and d). One of the important reasons for this was the good solubility of Sulfonamides. This indicated that Sulfonamides were more easily transported with soil solutions than other groups. Pig manure was the main contributor to the total antibiotic leaching into the groundwater in 2010 (Fig. 2c). In 2020, the total antibiotic leaching into groundwater was mainly from pig and sheep manure (Fig. 2d). However, there was large spatial variability among sub-basins (Supplementary Materials Fig. S18).

**Fig. 5:**
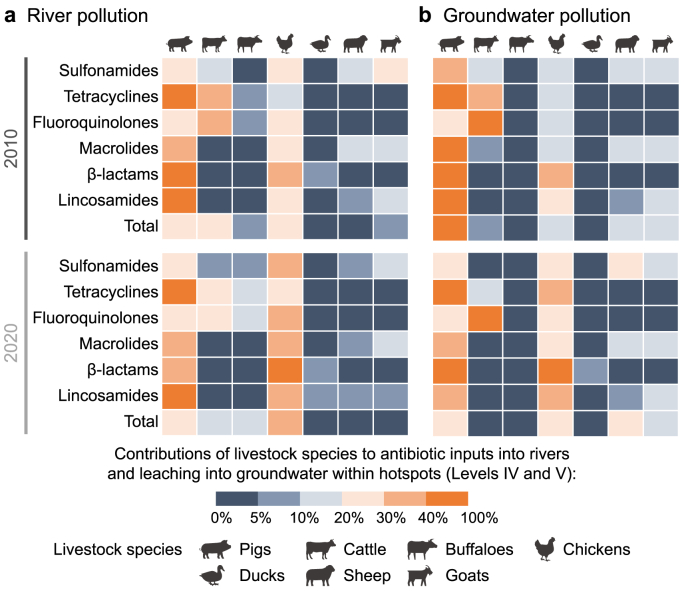
Shares of livestock species in river (**a**) and groundwater (**b**) pollution with antibiotics in hotspot sub-basins (Levels IV and V, %). Levels IV and V sub-basins are considered pollution hotspots (definition see Section 2.3). The shares of livestock species in the river and groundwater pollution in Levels I–III are in Supplementary Materials Fig. S8. Sources: the MARINA-Antibiotics (China-1.0) model (see Section 2.1 for the model description).

In Level I and II sub-basins, less than 0.2 g of antibiotics per km^2^ per year were leached into groundwater from livestock production in 2010 (Supplementary Materials Fig. S18). This resulted in approximately 1 tonne of antibiotics entering groundwater. Most of these sub-basins were located in southwestern China (Supplementary Materials Fig. S18), accommodated approximately 9% of the Chinese population, and covered 39% of the total sub-basin area in China (Supplementary Materials Figs. S10 and S11) in 2010. The share of the sub-basins in total groundwater pollution in China reached 32% in 2020 (Supplementary Materials Fig. S11). This implies that fewer sub-basins were identified as belonging to pollution Levels I–II in 2020 than in 2010. Between 2010 and 2020, changes in antibiotic leaching into groundwater from 2010 to 2020 ranged from −57% (decrease) to +147% (increase) among Level I–II sub-basins (Supplementary Materials Fig. S20). Some sub-basins in the southwest and northeast became cleaner in 2020 than in 2010 (Supplementary Materials Fig. S20). However, the northwest Level I and II Chinese sub-basins became more polluted in 2020 than in 2010 (Supplementary Materials Fig. S20). This was largely associated with the effects of climate change (lower surface runoff) and the relocation of livestock production activities (higher livestock density, Supplementary Materials Figs. S3–S5) in Level I and II sub-basins during 2010–2020. Sulfonamides constituted the predominant antibiotic group in the total antibiotic groundwater pollution in those sub-basins for both 2010 and 2020 (Supplementary Materials Fig. S12). Compared to 2010, the contributions of sheep manure to antibiotic groundwater pollution became more dominant in 2020 (Fig. 5; Supplementary Materials Fig. S13).

**Fig. 6:**
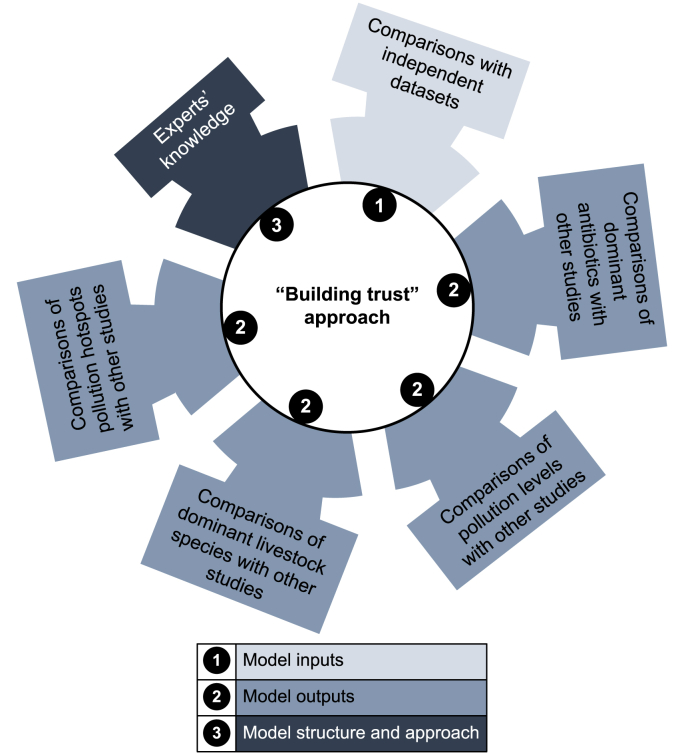
The conceptual framework for the ‘building trust’ approach for the large-scale water quality model. This framework is modified based on Strokal et al. [59]. Details on using the ‘building trust’ approach to evaluate the MARINA-Antibiotics (China-1.0) can be found in Section 3.3.

**Fig. 7:**
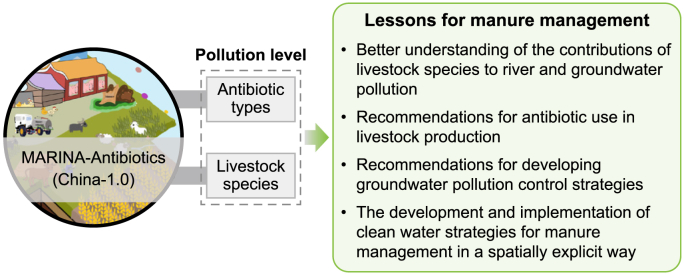
Overview of lessons from the MARINA-Antibiotics (China-1.0) model for manure management. Details can be found in Section 3.4.

Groundwater in Level III sub-basins received 4 tonnes of antibiotics in 2010 (Fig. 5). These sub-basins covered 52% of the national area, accommodated 58% of the total population in China (Supplementary Materials Figs. S10 and S11), and were mainly located in northeastern and southern China in 2010 (Supplementary Materials Fig. S18). By 2020, the total antibiotic leaching into groundwater increased by 28% in Level III sub-basins compared to that in 2010. As a result, more than 5 tonnes of antibiotics from livestock production leached into groundwater in Level III sub-basins in 2020. Approximately 64% of the total population lived in the Level III sub-basins in 2020. The decreases in antibiotic leaching into groundwater from 2010 to 2020 ranged from 28% to 48% among some southwestern and northeastern sub-basins (Supplementary Materials Fig. S20). These decreases were largely associated with climate change (more surface runoff) and the relocation of livestock species (reduced livestock production, Supplementary Materials Figs. S3–S5) in 2020. Most central and southern sub-basins experienced increased antibiotic leaching into groundwater, ranging from 25% to 161% (Fig. 5) by the year 2020. Sulfonamides were the dominant antibiotic group in the groundwater of the Level III sub-basins (Supplementary Materials Fig. S12). Compared to 2010, pigs and sheep remained the main contributors to antibiotic groundwater pollution in 2020 (Fig. 5, Fig. 6). The contributions of chicken and goat manure to groundwater pollution increased by 94% and 25% by 2020, respectively (Fig. 5, Fig. 6). These increases in antibiotic leaching into groundwater could be attributed to the combined effects of livestock production migration (increased livestock density of chicken and goat) and increased recycling of livestock manure as organic fertilizer (Supplementary Materials Figs. S3–S5 and Table S9) between 2010 and 2020 (see Fig. 7).

Approximately 10% of the Chinese sub-basin areas were identified as pollution hotspots for groundwater pollution (Levels IV and V) for the years 2010 and 2020 (Fig. 3). The hotspot sub-basins received 5 tonnes of total antibiotic leaching to groundwater in 2010. These sub-basins were in central and northern China. They accommodated around 31% of the total Chinese population in 2010 (Supplementary Materials Figs. S10–S11). The total antibiotic leaching into groundwater in hotspots nationally increased by 12% between 2010 and 2020 (Fig. 2, Fig. 5). This resulted in 6 tonnes of antibiotic leaching into groundwater in hotspot sub-basins in 2020. By 2020, changes in antibiotic leaching into groundwater were estimated to vary from −69% (decrease) to +70% (increase) for hotspot sub-basins (Fig. 5). In 2010, pigs were the dominant contributor to antibiotic contamination of groundwater in the hotspots (Fig. 6). Between 2010 and 2020, the contributions of chicken and sheep manure to the total antibiotic leaching into groundwater increased by 40% and 67%, respectively (Fig. 5, Fig. 6). Sulfonamides remained the main antibiotic group in groundwater pollution in 2020. In 2020, β-lactams from chickens, pigs, and ducks accounted for more than 10% of the total antibiotics leaching into groundwater (Fig. 5; Supplementary Materials Fig. S12).

### 3.3 Model evaluation, limitations, and uncertainties

We applied four model evaluation options following a widely used approach to build trust in the models [10,59] (see Section 2.2). For Option 1, we compared the following model inputs with other datasets: soil pH, soil temperature, soil organic carbon content, and soil saturation. The selected model inputs were plotted on a 1:1 line. We assessed the model performance using two statistical indicators: Pearson’s coefficient of determination (*R*_P_^2^, from 0 to 1) and the Nash-Sutcliffe efficiency (NSE, from −∞ to 1). According to the performance rates from Moriasi et al. [61], the differences between our model inputs and independent datasets were acceptable: *R*_P_^2^ > 0.8 and NSE >0.6 (see details in Supplementary Materials Figs. S14–S16).

Option 2 showed that our model outputs for Tetracyclines, Fluoroquinolones, and Sulfonamides as the dominant contributors to antibiotic pollution in rivers in China were in line with those of existing studies [1,2,32]. We modeled that pig manure contributed approximately 1106 and 517 tonnes of antibiotic inputs to the Yangtze River basins in 2010 and 2020, respectively. Chen et al. [62] used the level Ⅳ fugacity model to evaluate the emission, multimedia fate, and risk of antibiotics in the entire Yangtze River basin. Their model results indicated that, between 2013 and 2021, approximately 514–903 tonnes of antibiotics were introduced into the Yangtze River from pig production. Our model results were slightly higher than those of their study because we were more complete regarding the number of antibiotics used (24 in our study and 18 in Chen et al. [62]). Zhang et al. [1] also indicated that pigs accounted for over 40% of river antibiotic inputs in 2013. This was demonstrated by our main livestock species responsible for antibiotic river pollution in 2010.

Option 3 focused on comparing the spatial variability of pollution hotspots with those of other studies [1,2,32,63]. For instance, the river pollution hotspots in central and northern China in 2010 and 2020 were in line with the findings of previous studies [1,2,32]. Generally, these sub-basins (e.g., Hai and Yangtze River sub-basins) had high livestock densities (Supplementary Materials Figs. S3–S5) and high manure inputs per km^2^ (Supplementary Materials Fig. S10) both in 2010 and 2020. This was in line with previous studies (e.g., Refs. [2,32,63,64]). Other studies have shown that areas with higher direct manure discharges received more pollutants from rivers in 2010 [10]. This was also demonstrated by our hotspot sub-basins in 2010. Examples were sub-basins in southern China (e.g., Yangtze River sub-basins) with higher direct discharges of livestock manure to rivers than those in northern China (e.g., Songhua River sub-basins) in 2010. Other studies have indicated that antibiotics in rivers were generally higher in central and eastern China than in western China [2,3,65]. Huang et al. [2] indicated that antibiotic groundwater pollution was higher in the Hai River Basin than in the Yangtze and Pearl River Basins, which was consistent with our study for both 2010 and 2020.

For Option 4, we reflected on our modeling approach. Our model was an integrated modeling approach that combined the existing knowledge and literature on the soil processes of antibiotics that can be transported to rivers and groundwater. First, we reviewed the literature on the physical, biological, and chemical processes that can affect the degradation and adsorption of antibiotics in the soil and soil erosion [40,41,50,51]. We developed an approach for the degradation and adsorption of antibiotics in soil particles and solutions based on expert knowledge of field experiments. We also used expert knowledge of soil erosion supported by literature to estimate the antibiotics in soil particles transported from agricultural land to rivers. The four aforementioned options helped us to better understand the performance of our model (inputs, outputs, and approach) and to better interpret the results.

However, our model did not account for sources such as antibiotics in sewage systems or antibiotic manufacturing and processing. Thus, the river and groundwater pollution levels may have been underestimated. Extreme events (e.g., heavy rainfall) were not considered, which may have resulted in more antibiotics entering rivers through runoff and soil erosion and leaching into groundwater at certain moments. We focused on 2010 and 2020. Thus, the trends in our pollution levels during 2010 and 2020 may not consistently decrease but could initially increase and then decrease. Our model results were under- or overestimated in sub-basins, depending on their agricultural development. Future studies may need to quantify the processes and dynamics of antibiotic inputs to rivers and groundwater in more detail and consider the by-products that emerge during degradation processes for more years. However, this study focused on livestock and manure production. We quantified the flows of 24 antibiotics from livestock into rivers and groundwater. We considered important sources of antibiotics in livestock production: pigs, cattle, chickens, ducks, goats, sheep, and buffaloes. Our selection of seven livestock species was justified by three main reasons. First, these livestock species are of high economic importance for the whole of China [7,55,66,67]. The selected livestock species contributed largely to China’s agricultural production and food systems [7,55]. These livestock species could reflect those most widely produced across the whole of China, thus covering diverse production systems [10]. Second, livestock species with potentially varying environmental impacts were included to explore how different manure management systems (e.g., storage vs. grazing) affected inputs of pollutants into rivers and leaching into groundwater between 2010 and 2020 [8,10,68]. Third, we recognized that the main livestock species may vary geographically, as in northern vs. southern China, where environmental and agricultural factors influenced species prevalence [55]. However, the selections of these seven livestock species reflected the broader national significance rather than focusing on region-specific species. They were important across multiple regions, even though their relative importance varied by location. Thus, the uncertainties associated with missing sources did not affect our conclusions. Future studies could use our modeling tool and add missing sources to better understand the contribution of other sources to antibiotic pollution.

In this study, we developed a steady-state, large-scale water quality model to estimate antibiotic loadings input into rivers and leaching into groundwater at the sub-basin scale. We estimated loadings of antibiotics into waters (not concentrations). Our mode did not consider local-scale factors, such as the exact distance between farms and nearby rivers. The current model inputs were all at the sub-basin scale. Thus, our model may not be directly used for local analyses (e.g., specific farms or watersheds). However, our modeling approach was integrated and more process-oriented than the existing models [1,17,62]. Our model was run annually to examine changes in river and groundwater pollution from antibiotics in livestock production in 2010 and 2020 at the sub-basin scale. Our model can also be used for other years if data are available. We considered the different pathways that contribute to antibiotics in rivers and groundwater, including surface runoff, soil erosion, and leaching. Seven dominant livestock species in China were considered. Our approach can simultaneously quantify antibiotic river and groundwater pollution from 24 antibiotics and seven livestock species, which has not been done previously for over 300 sub-basins in China. Because our model was integrated, process-based, and uncalibrated, it offers an opportunity to conduct future analyses and account for climate changes, technological developments, and food production drivers. All datasets used in our study were widely used and accepted by the scientific community, and they were freely available for download [1,[18], [19], [20], [21], [22],^25^,^44^,^57^,^69^].

### 3.4 Lessons from water quality modeling for manure management

Our analyses drew four lessons regarding antibiotic pollution in rivers and groundwater. First, our study helped identify the contributions of livestock species to river and groundwater pollution in 2010 and 2020. This study was conducted in China. We showed that pig and cattle manure were the dominant contributors to river pollution with antibiotics in 2010 (Fig. 2, Fig. 3, Fig. 4, Fig. 5). In 2020, pig and chicken production became the dominant sources of antibiotic-related river pollution. Other countries can use our new modeling tool and increase their understanding of the species and efforts required to reduce livestock-production-related river and groundwater pollution. From our analyses, we learned that, for non-hotspot sub-basins (Levels I–III), a reduction of water pollution with antibiotics was needed from pig, sheep, and cattle production. For hotspot sub-basins (Levels IV–V), future water pollution strategies should focus more on managing chicken and pig manure. This information supported the formulation of livestock-specific manure management. Our model can be a useful tool for projecting future antibiotic rivers and groundwater and supporting decision-makers for specific livestock species that would be prioritized in manure management policies in the future.

Second, our study provided a better understanding of the antibiotic groups from livestock production toward rivers and groundwater. We showed that Sulfonamides were important for river pollution in Level I sub-basins, and this pollution resulted mainly from the manure of sheep and cattle, both in 2010 and 2020. Fluoroquinolones and Sulfonamides were the most important river pollutants in Levels II–V sub-basins and mainly originated from chicken and pig manure in 2020. This study focused on seven livestock species due to their economic significance (e.g., most widely farmed and consumed livestock in China [8,55,67]) and data availability. Future research can consider expanding to include more livestock species based on our model. We also realized that, in reality, there may be even more antibiotics present in Chinese waters, but this model can be easily extended to other antibiotics. In other countries, other antibiotics may dominate in the use for livestock; for example, Tetracyclines were predominantly used in livestock production in the United States [70], Cyprus, Bulgaria, and Portugal [71], whereas in Australia, Macrolides were the main antibiotic groups used in livestock production [72]. Applying our modeling tool could help those countries understand which livestock species can contribute to water pollution.

Third, our results supported existing agricultural policies for better groundwater and river pollution control with other pollutants in China. Our results indicated less antibiotic river pollution and more groundwater pollution during 2010–2020 in China as a whole (Fig. 2, Fig. 4, Fig. 5). Manure contains other pollutants, such as nutrients and pathogens [10]. With the implications of agricultural policies, for example, ‘Livestock and Poultry Manure Utilization Action Plan (2017–2020)’ [34], ‘14th Five-Year National Agricultural Green Development Plan’ [33], and China’s livestock relocation policies [36], etc.), more manure recycling on agricultural land may not only affect water pollution with antibiotics in China as we estimated in our study. These may also reduce nutrient or pathogen pollution in rivers. This has also been demonstrated by existing studies on other pollutants (e.g., nutrients). Recycling manure was shown to be the most cost-effective option for reducing future coastal eutrophication [73]. However, there may be a trade-off between policies facilitating more manure recycling and groundwater pollution, as indicated by our study's changes in antibiotic groundwater pollution (Fig. 5; Supplementary Materials Fig. S20). This implies that future agricultural policies are required to avoid the trade-off between the recycling of manure on land and groundwater pollution.

Fourth, our results provided policymakers with a better understanding of the development and implementation of clean water strategies for manure management in a spatially explicit manner. Sub-basin analyses can help identify pollutants' origin and sources to formulate effective solutions for agriculture-related pollution. Our model results showed that antibiotic river pollution in some sub-basins (e.g., northern sub-basins in China, Supplementary Materials Figs. S19–S20) increased, whereas in others (e.g., southern sub-basins in China) decreased from 2010 to 2020. This may be associated with increased livestock production in China’s northern and southern sub-basins between 2010 and 2020. This implied that future manure management policies for these sub-basins need to be combined with better treatment to avoid more pollutants in rivers. We also found that avoiding the direct discharges of manure could considerably decrease antibiotic inputs to rivers in the southern sub-basins of China. However, our results indicated that antibiotic leaching into groundwater increased by 11% from 2010 to 2020 in these southern sub-basins. This implied the importance of considering the potential trade-off between manure recycling and groundwater for developing future water pollution controls. As we indicated in the Introduction section, our study could raise the attention of policymakers, the public, and other stakeholders on the importance of considering antibiotics in national water quality policies and monitoring programs in the future.

## 4 Conclusions

This study was the first attempt to account for antibiotics from livestock production in rivers and groundwater in China at the sub-basin scale. The MARINA-Antibiotics (China-1.0) model was developed and evaluated to quantify the flow of 24 antibiotics into rivers and leaching into groundwater from seven livestock species in 395 Chinese sub-basins and to examine changes in antibiotic water pollution between 2010 and 2020. In 2010 and 2020, 8364 and 3436 tonnes of antibiotics entered rivers and groundwater, respectively, causing antibiotic pollution. 50–90% of the antibiotic losses to rivers and groundwater originated from 40% of the basin areas in China between 2010 and 2020. The total river antibiotic inputs decreased by 59% during 2010–2020 because of fewer manure point sources. In contrast, total antibiotic leaching into groundwater increased by 15% nationally, which was largely because of increased manure recycling. Fluoroquinolones were responsible for 55% of the antibiotics in Chinese rivers in 2010 and 2020 and mainly originated from pigs, cattle, and chicken manure. Sulfonamides were responsible for over 90% of groundwater antibiotics, mainly from pig and sheep manure. Our study supports future agriculture-related policy designs in China.

## CRediT authorship contribution statement

**Qi Zhang:** Writing - Original Draft, Visualization, Validation, Methodology, Formal Analysis, Data Curation, Conceptualization. **Yanan Li:** Writing - Review & Editing, Methodology, Conceptualization. **Carolien Kroeze:** Writing – Review & Editing, Supervision, Conceptualization. **Milou G.M. van de Schans:** Writing - Review & Editing, Methodology. **Jantiene Baartman:** Writing - review & Editing, Methodology. **Jing Yang:** Writing - Review & Editing, Investigation. **Shiyang Li:** Writing - Review & Editing, Investigation. **Wen Xu:** Writing - Review & Editing, Supervision. **Mengru Wang:** Writing - Review & Editing. **Lin Ma:** Writing - Review & Editing, Supervision. **Fusuo Zhang:** Writing - Review & Editing, Supervision. **Maryna Strokal:** Writing - Review & Editing, Supervision, Methodology, Conceptualization.

## Data availability

All sources to model inputs are provided in the Supplementary Materials. The model is processed in the R script. Model outputs are presented in the manuscript and the Supplementary Materials.

## Declaration of competing interest

The authors declare that they have no known competing financial interests or personal relationships that could have appeared to influence the work reported in this paper.
