# Supplementary material for: More inputs of antibiotics into groundwater but less into rivers as a result of manure management in China
Source: Environ Sci Ecotechnol. 2024 Nov 26;23:100513. doi: 10.1016/j.ese.2024.100513 (PMC11697712; doi:10.1016/j.ese.2024.100513)
Supplement: Multimedia component 1 [file mmc1.docx]

**Supplementary Materials:** **More inputs of antibiotics into groundwater but less into rivers as a result of manure management in China**

*Qi Zhang ^a,b,*^, Yanan Li ^a,b^, Carolien Kroeze^b^, Milou G.M. van de Schans ^c^, Jantiene Baartman ^d^, Jing Yang ^e^, Shiyang Li ^d^, Wen Xu^a,*^, Mengru Wang ^b^, Lin Ma ^e^, Fusuo Zhang ^a^, Maryna Strokal ^b,*^*

^a^ College of Resources and Environmental Sciences; National Academy of Agriculture Green Development; Key Laboratory of Plant-Soil Interactions, Ministry of Education, China Agricultural University, 100193, China.

^b^ Earth Systems and Global Change group, Environmental Sciences Department, Wageningen University & Research, Droevendaalsesteeg 4, Wageningen, 6708 PB, The Netherlands.

^c^ Wageningen Food Safety Research, Wageningen University and Research, Akkermaalsbos 2, 6708 WB, Wageningen, The Netherlands.

^d^ Soil Physics and Land Management Group, Wageningen University & Research, Droevendaalsesteeg 3, Wageningen, 6708 PB, The Netherlands.

^e^ Key Laboratory of Agricultural Water Resources, Hebei Key Laboratory of Soil Ecology, Center for Agricultural Resources Research, Institute of Genetic and Developmental Biology, The Chinese Academy of Sciences, Hebei, 050021, China.

^f^ Interdisciplinary Research Center for Agriculture Green Development in Yangtze River Basin, Southwest University, Tiansheng Road 02, Chongqing 400715, China.

*Correspondence: [qi.zhang@wur.nl](mailto:qi.zhang@wur.nl) (Q.Z.), [maryna.strokal@wur.nl](mailto:maryna.strokal@wur.nl) (M.S.); [wenxu@cau.edu.cn](mailto:wenxu@cau.edu.cn) (W.X.)

**Supplementary Materials Text:** MARINA-Antibiotics model

**Figures in Supplementary Materials**

**Fig. S1.** The classification of antibiotics and their groups.

**Fig. S2.** Sources of the model inputs for the MARINA-Antibiotics model (**Model to Assess River Inputs of pollutaNts to seAs** for Antibiotics in freshwater).

**Fig. S3.** Livestock numbers in 395 sub-basins in the year 2010 (LSU/year).

**Fig. S4.** Livestock numbers in 395 sub-basins in the year 2020 (LSU/year).

**Fig. S5.** Total livestock numbers in 395 sub-basins in the years 2010 and 2020 (LSU/year).

**Fig. S6.** Shares of human population living in sub-basins with pollution levels I-V for antibiotics in rivers in the years 2010 and 2020 (% share of the total national population).

**Fig. S7.** Shares of sub-basin area with pollution levels I-V for antibiotics in rivers in the years 2010 and 2020 (% share of total surface drainage area).

**Fig. S8.** Shares of livestock species in river pollution with antibiotics in Levels I-III sub-basins (%).

**Fig. S9.** Shares of antibiotic groups in river pollution in Levels I-V sub-basins (%).

**Fig. S10.** Shares of human population living in sub-basins with pollution levels I-V for antibiotics leaching to groundwater in the years 2010 and 2020 (% share of the total national population).

**Fig. S11.** Shares of sub-basin area with pollution levels I-V for antibiotics leaching to groundwater in the years 2010 and 2020 (% share of total surface drainage area).

**Fig. S12.** Shares of antibiotic groups in groundwater pollution in Levels I-V sub-basins (%).

**Fig. S13.** Shares of individual antibiotic groups in the total inputs to groundwater in sub-basins that belong to pollution levels I-III (%).

**Fig. S14.** Model validation of soil organic carbon content input data for the MARINA-Antibiotics model.

**Fig. S15.** Model validation of soil saturation input data for the MARINA-Antibiotics model.

**Fig. S16.** Model validation of soil pH input data for the MARINA-Antibiotics model.

**Fig. S17.** Model validation of soil temperature input data for the MARINA-Antibiotics model.

**Fig. S18.** Antibiotic pollution in waters from livestock production at the sub-basin scale in China in 2010 and 2020 (g antibiotics/km^2^/year).

**Fig. S19.** Changes in antibiotic inputs into rivers at sub-basin scale between 2010 and 2020 (%).

**Fig. S20.** Changes in antibiotic leaching into groundwater at sub-basin scale between 2010 and 2020 (%).

**Tables in Supplementary Materials**

**Table S1.** Equations of the MARINA-Antibiotics model to quantify manure-associated inputs of antibiotics to rivers and groundwater from grazing and storage systems in 395 Chinese sub-basins.

**Table S2.** Descriptions of the abbreviations in Table S1.

**Table S3.** Descriptions of how model inputs are processed to sub-basins for the MARINA-Antibiotics model (**M**odel to **A**ssess **R**iver Inputs of polluta**N**ts to se**A**s for Antibiotics in freshwater).

**Table S4.** Livestock unit coefficients in China that are derived from Eurostat.

**Table S5.** Antibiotics for use in human medicine and food livestock production.

**Table S6.** Antibiotics in rivers resulted from diffuse sources (surface runoff and soil erosion) in northern and southern sub-basins in China between 2010 and 2020 (tonnes/year).

**Table S7.** Antibiotics in rivers resulted from point sources in northern and southern sub-basins in China between 2010 and 2020 (tonnes/year).

**Table S8.** Antibiotics leaching to groundwater in northern and southern sub-basins in China between 2010 and 2020 (tonnes/year).

**Table S9.** Antibiotics in livestock manure applied on land in northern and southern sub-basins in China between 2010 and 2020 (tonnes/year).

**Table S10.** Range of the model parameter values for antibiotic inputs into rivers and leaching into groundwater (0-1).

**Supplementary Materials Text: MARINA-Antibiotics model**

We developed the MARINA-Antibiotics model. MARINA is short for the Model to Assess River Inputs of pollutaNts to seAs for Antibiotics in freshwater. MARINA-Antibiotics quantifies the annual inputs of antibiotics to rivers and groundwater from livestock manure at the sub-basin scale in China in the years 2010 and 2020. Table S1 provides the main equations of the MARINA-Antibiotics model. Inputs of antibiotics to rivers and groundwater are calculated as a function of livestock numbers, excretion rates of antibiotics in manure, manure production, manure management, the degradation (persistence) in manure during storage, degradation and sorption in the soils, runoff, erosion and leaching. There are four steps to calculate antibiotics from manure in rivers and leaching to groundwater. First, excreted antibiotics in manure from livestock species are calculated (Table S1). Second, inputs of antibiotics to agricultural land from manure application are calculated. This includes manure from storage and grazing systems. Third, inputs of antibiotics from land to rivers are calculated (see Fig. 1 in the main manuscript, Tables S1-S4 in the Supplementary Materials). Inputs of antibiotics in rivers result from diffuse and point sources. Diffuse sources are inputs of antibiotics from land (applied manure with antibiotics on agricultural land) to rivers via runoff and soil erosion. Point sources are inputs of antibiotics to rivers from the direct discharges of manure during storage systems (Fig. 1 in the manuscript). Fourth, antibiotic leaching to groundwater is calculated (Tables S1-S4).


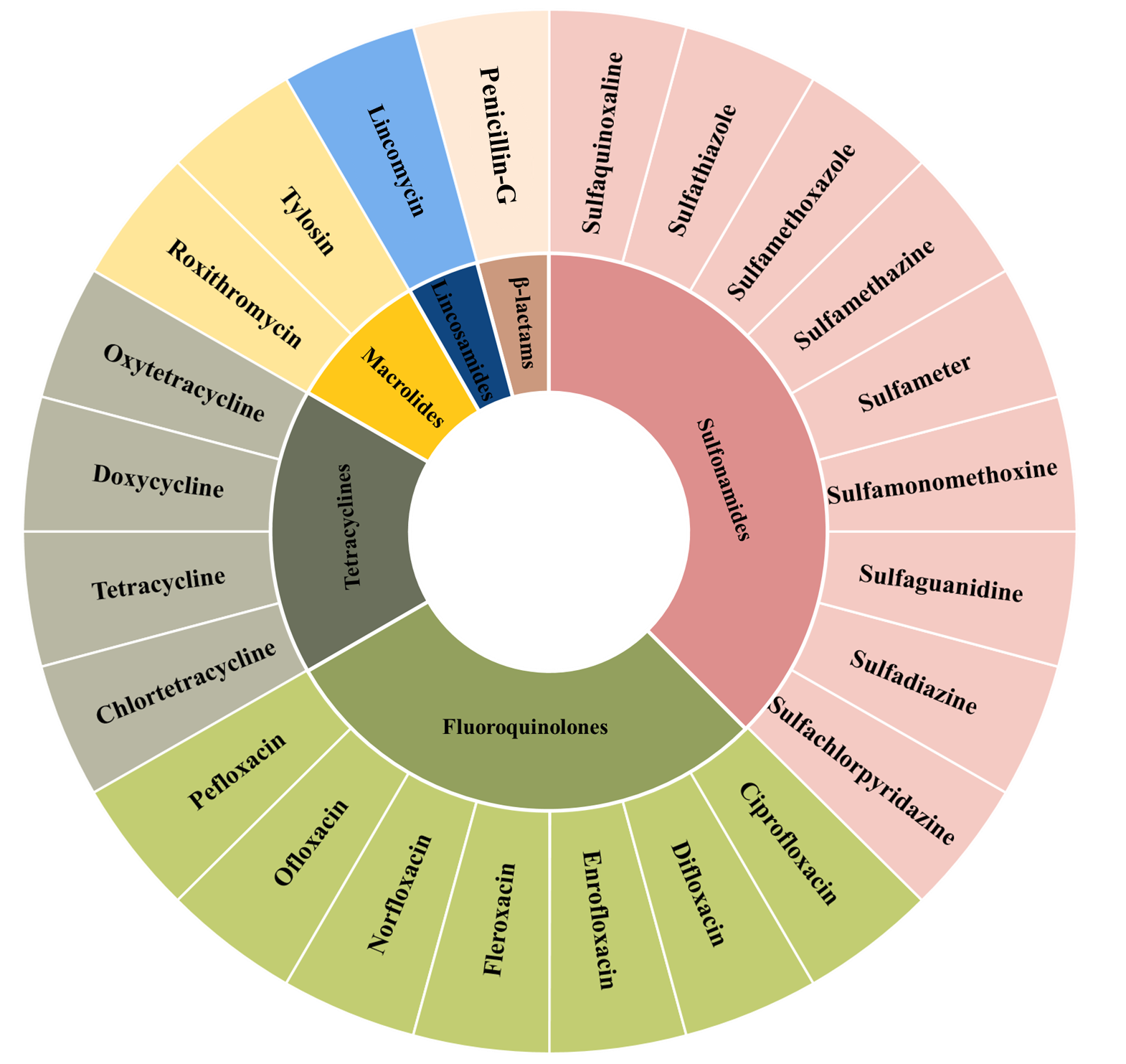


**Fig. S1. The classification of antibiotics and their groups.** Our study focuses on six groups of antibiotics including (1) Sulfonamides, (2) Tetracyclines, (3) Fluoroquinolones, (4) Macrolides, (5) β-lactams, and (6) Lincosamides. The Sulfonamides group (pink colors) is subdivided into nine subgroups, which are Sulfaquinoxaline, Sulfathiazole, Sulfamethoxazole, Sulfamethazine, Sulfameter, Sulfamonomethoxine, Sulfaguanidine, Sulfadiazine, and Sulfachlorpyridazine. The Tetracyclines group (darker green colors) is subdivided into four subgroups, which are Oxytetracycline, Doxycycline, Tetracycline, and Chlortetracycline. The Fluoroquinolones group (light green colors) is subdivided into seven subgroups, which are Pefloxacin, Ofloxacin, Norfloxacin, Fleroxacin, Enrofloxacin, Difloxacin, Ciprofloxacin. The Macrolides group (yellow colors) is subdivided into two subgroups, which are Tylosin and Roxithromycin. The other groups are β-lactams (Penicillin-G in brown colors) and Lincosamides (Lincomycin in blue colors).

**
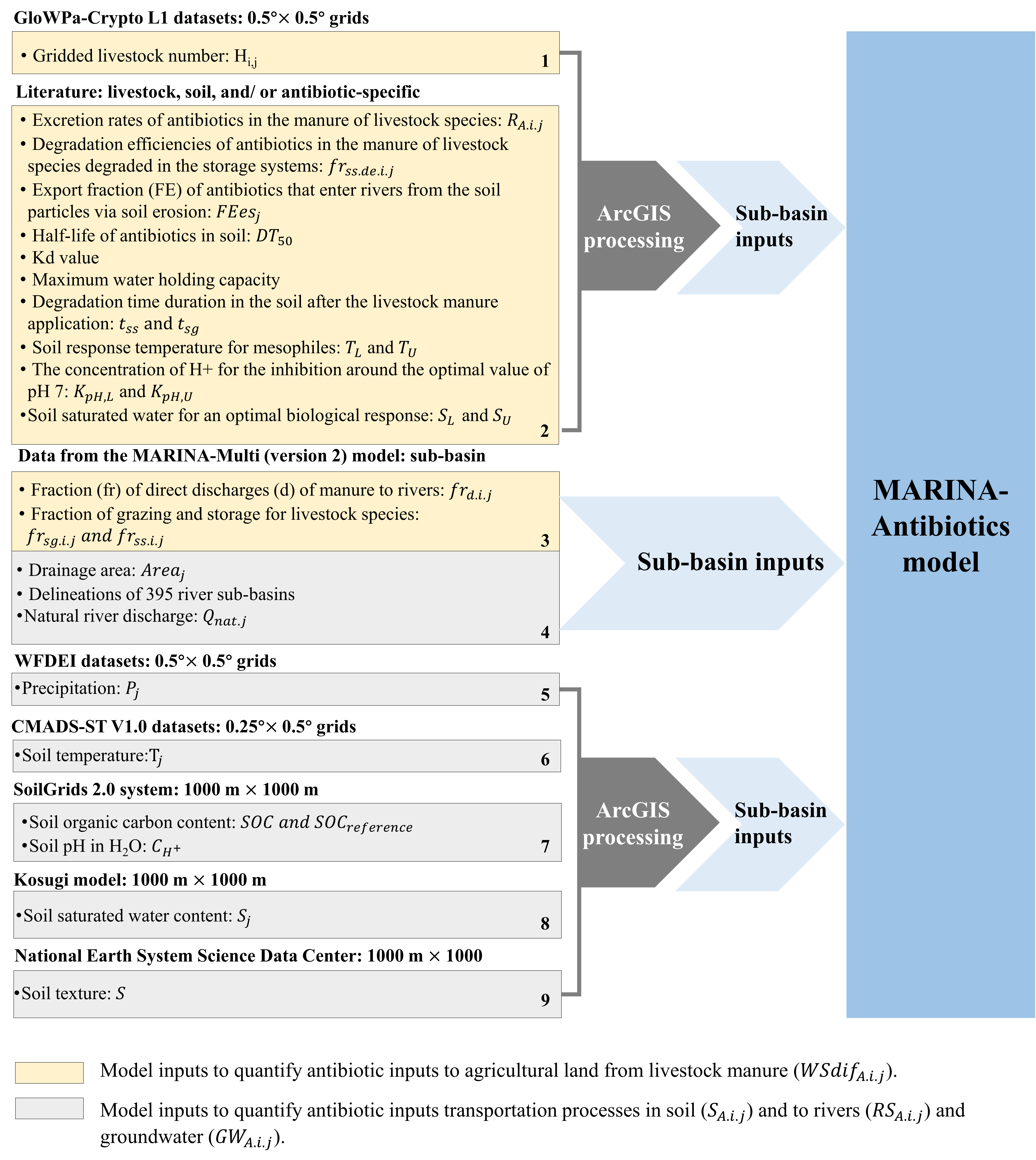
**

**Fig. S2.** **Sources of the model inputs for the MARINA-Antibiotics model** (Model to Assess River Inputs of pollutaNts to seAs for Antibiotics in freshwater). References to the sources are shown in Table S3. Numbers 1-9 indicate model input categories (see Table S3).

**Fig. S3. Livestock numbers in 395 sub-basins in the year 2010 (LSU/year).** LSU is short for livestock units (see details in Table S5). Livestock species include buffaloes, cattle, pigs, chickens, ducks, goats, and sheep. This information is derived from Li, et al. [1] and Vermeulen, et al. [2] and aggregated to 395 sub-basins for the MARINA-Antibiotics model. Sources: the MARINA-Antibiotics model (see Section 2.1 for the model description in the main manuscript).

**Fig. S4. Livestock numbers in 395 sub-basins in the year 2020 (LSU/year).** LSU is short for livestock units (see details in Table S5). Livestock species include buffaloes, cattle, pigs, chickens, ducks, goats, and sheep. Chinese statistic yearbooks from 2010 [3] and 2020 [4] are used to update the livestock number and spatial distribution of livestock species and aggregated to 395 sub-basins for the MARINA-Antibiotics mode in 2020. Sources: the MARINA-Antibiotics model (see Section 2.1 for the model description in the main manuscript).

**Fig. S5. Total livestock numbers in 395 sub-basins in the years 2010 and 2020 (LSU/year). LSU is short for livestock units (see details in Table S5).** The right maps show the spatial distributions of livestock numbers in 2010 and 2020. The left map shows the spatial distribution of changes in total livestock numbers between 2010 and 2020. Livestock species include buffaloes, cattle, pigs, chickens, ducks, goats, and sheep. Sources: the MARINA-Antibiotics model (see Section 2.1 for the model description in the main manuscript).


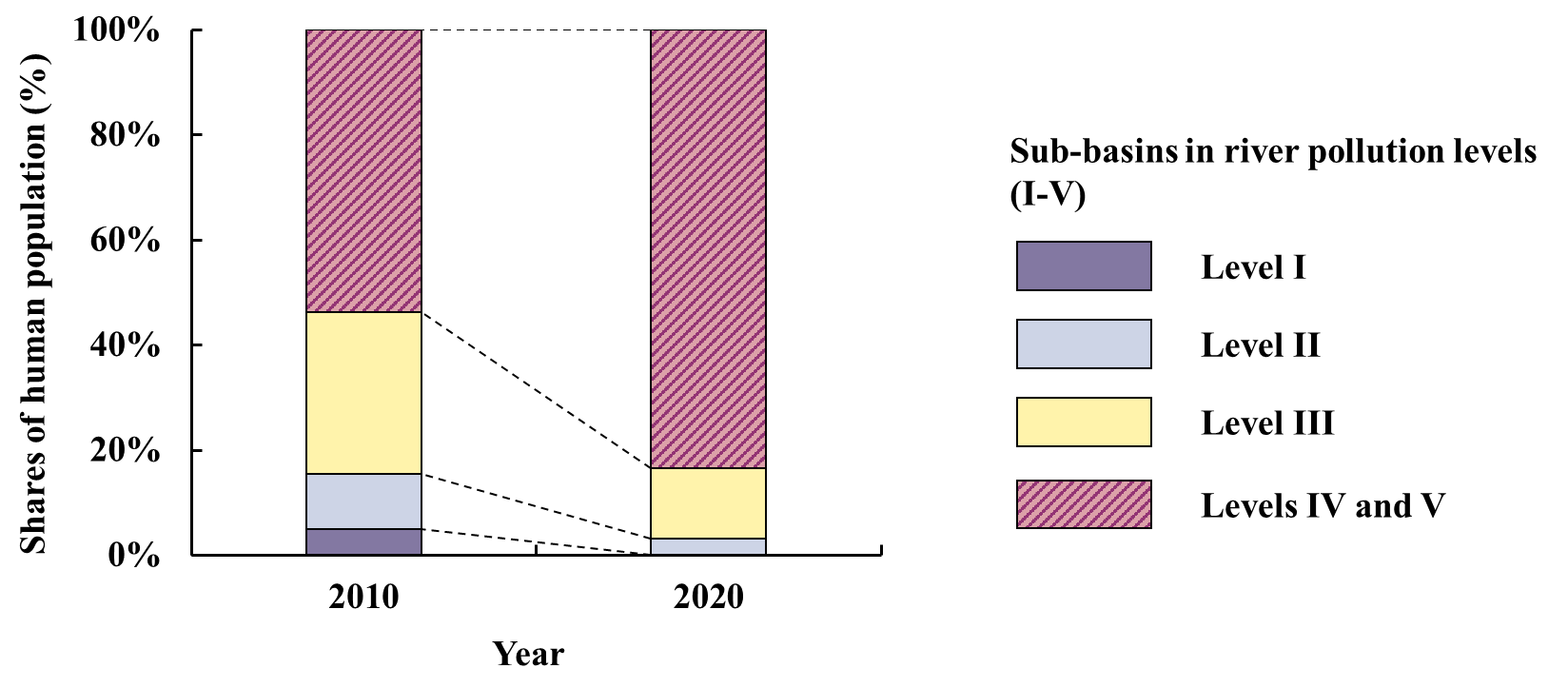
**Fig. S6. Shares of human population living in sub-basins with pollution levels I-V for antibiotics in rivers in the years 2010 and 2020 (% share of the total national population).** Sources: the MARINA-Antibiotics model (see Section 2.1 for the model description in the main manuscript). Levels I-V refer to the pollution levels of total antibiotic inputs to rivers (definition see Section 2.3 in the main manuscript).


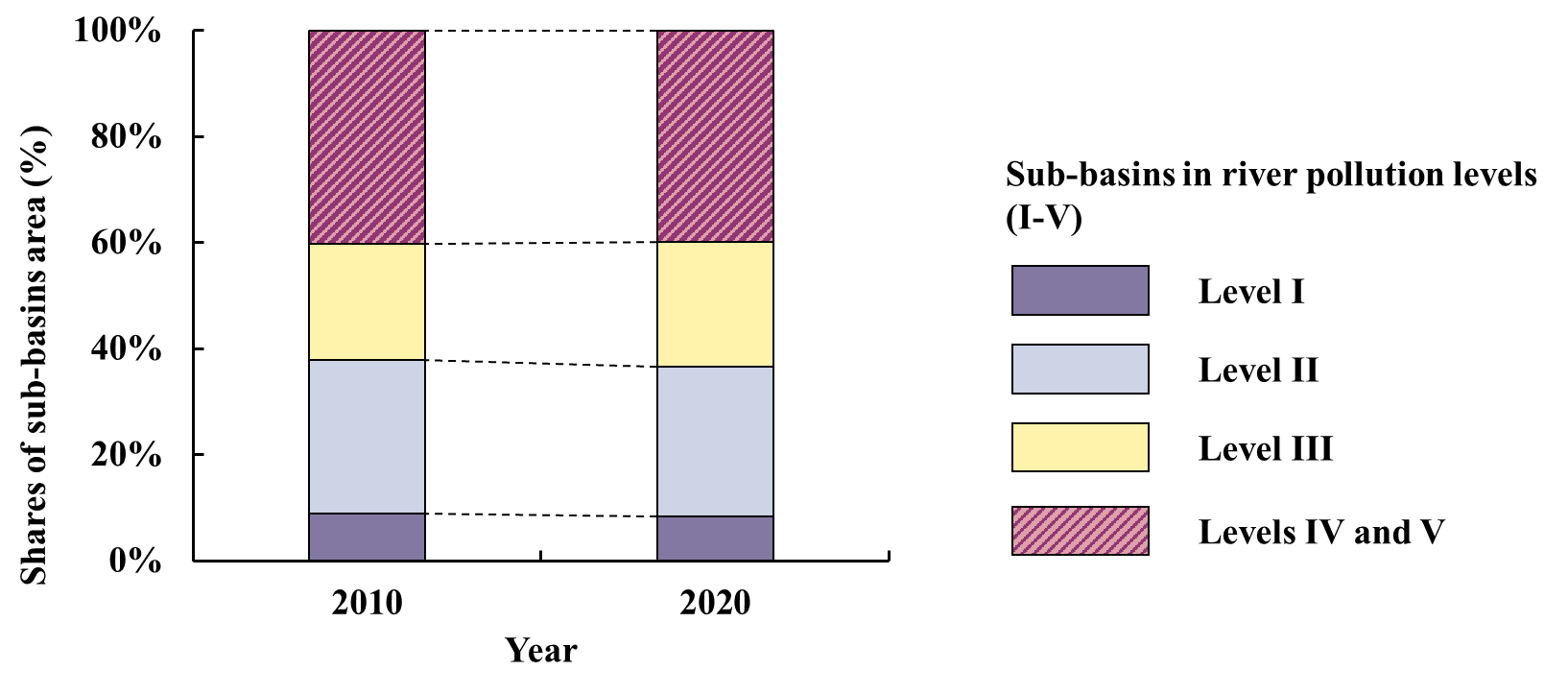


**Fig. S7. Shares of sub-basin area with pollution levels I-V for antibiotics in rivers in the years 2010 and 2020 (% share of total surface drainage area).** Sources: the MARINA-Antibiotics model (see Section 2.1 for the model description in the main manuscript). Levels I-V refer to the pollution levels of total antibiotic inputs to rivers (definition see Section 2.3 in the main manuscript).


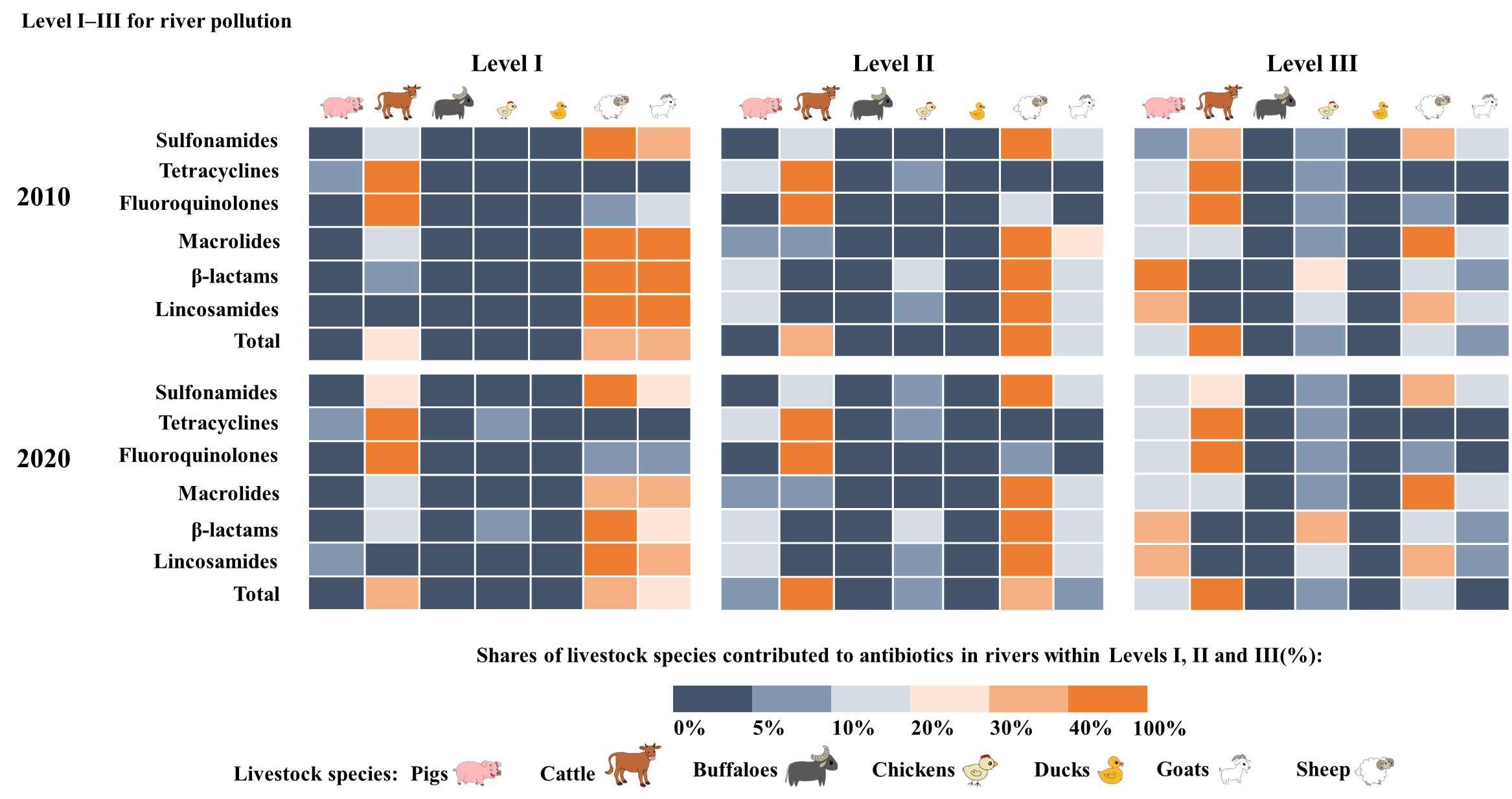


**Fig. S8. Shares of livestock species in river pollution with antibiotics in Levels I-III sub-basins (%).** Sources: the MARINA-Antibiotics model (see Section 2.1 for the model description in the main manuscript). Levels I-III refer to the pollution levels of total antibiotic inputs to rivers (definition see Section 2.3 in the main manuscript).


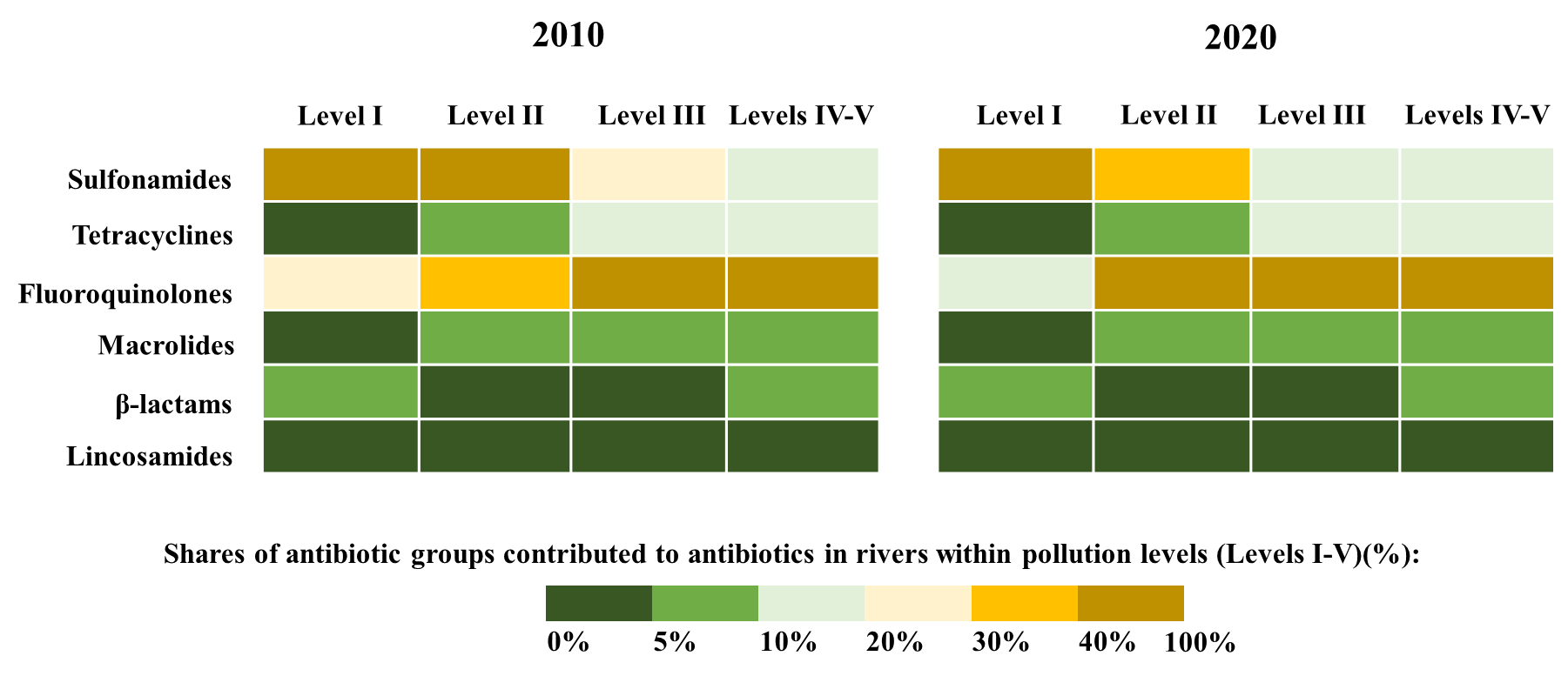


**Fig. S9. Shares of antibiotic groups in river pollution in Levels I-V sub-basins (%).** Sources: the MARINA-Antibiotics model (see Section 2.1 for the model description in the main manuscript). Levels I-III refer to the pollution levels of total antibiotic inputs to rivers (definition see Section 2.3 in the main manuscript).


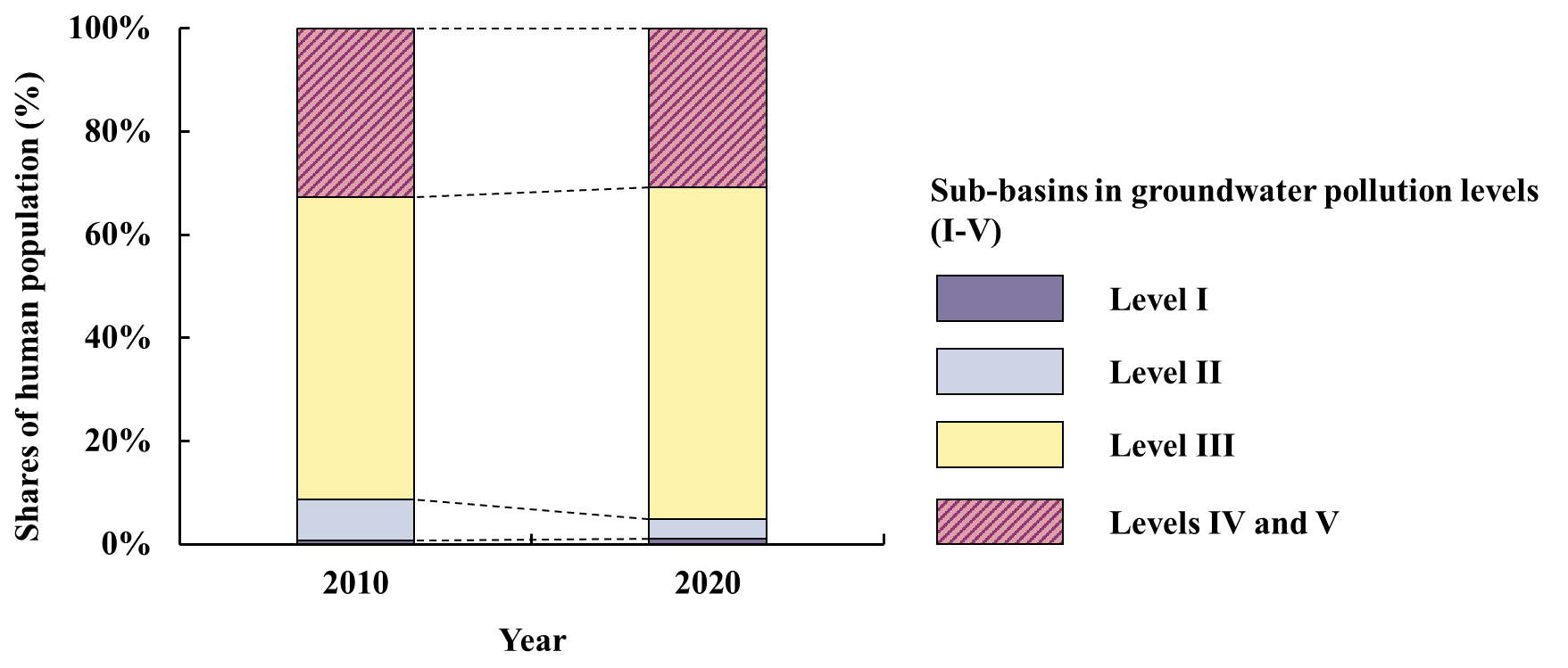
**Fig. S10. Shares of human population living in sub-basins with pollution levels I-V for antibiotics leaching to groundwater in the years 2010 and 2020 (% share of the total national population).** Sources: the MARINA-Antibiotics model (see Section 2.1 for the model description in the main manuscript). Levels I-V refer to the pollution levels of total antibiotic leaching to groudnwater (definition see Section 2.3 in the main manuscript).


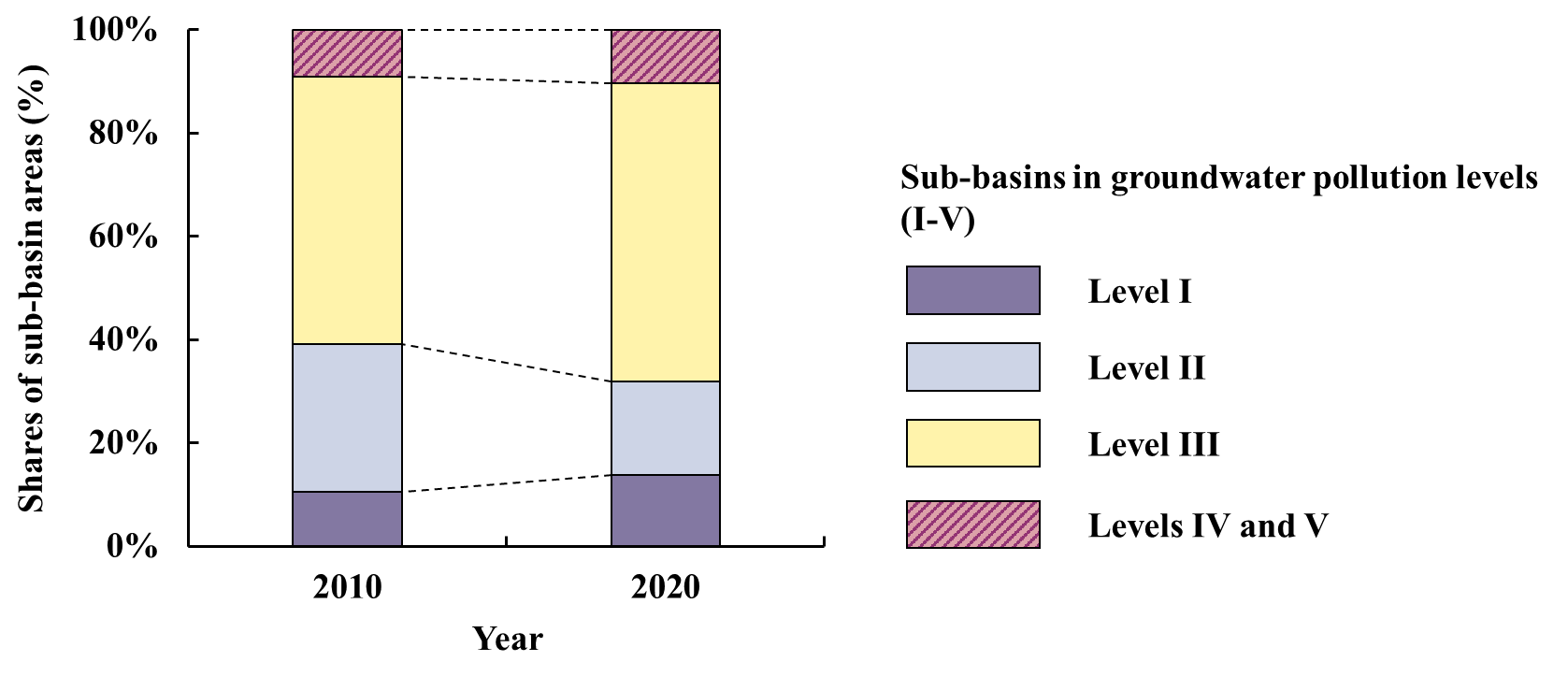


**Fig. S11. Shares of sub-basin area with pollution levels I-V for antibiotics leaching to groundwater in the years 2010 and 2020 (% share of total surface drainage area).** Sources: the MARINA-Antibiotics model (see Section 2.1 for the model description in the main manuscript). Levels I-V refer to the pollution levels of total antibiotic leaching to groundwater (definition see Section 2.3 in the main manuscript).

**
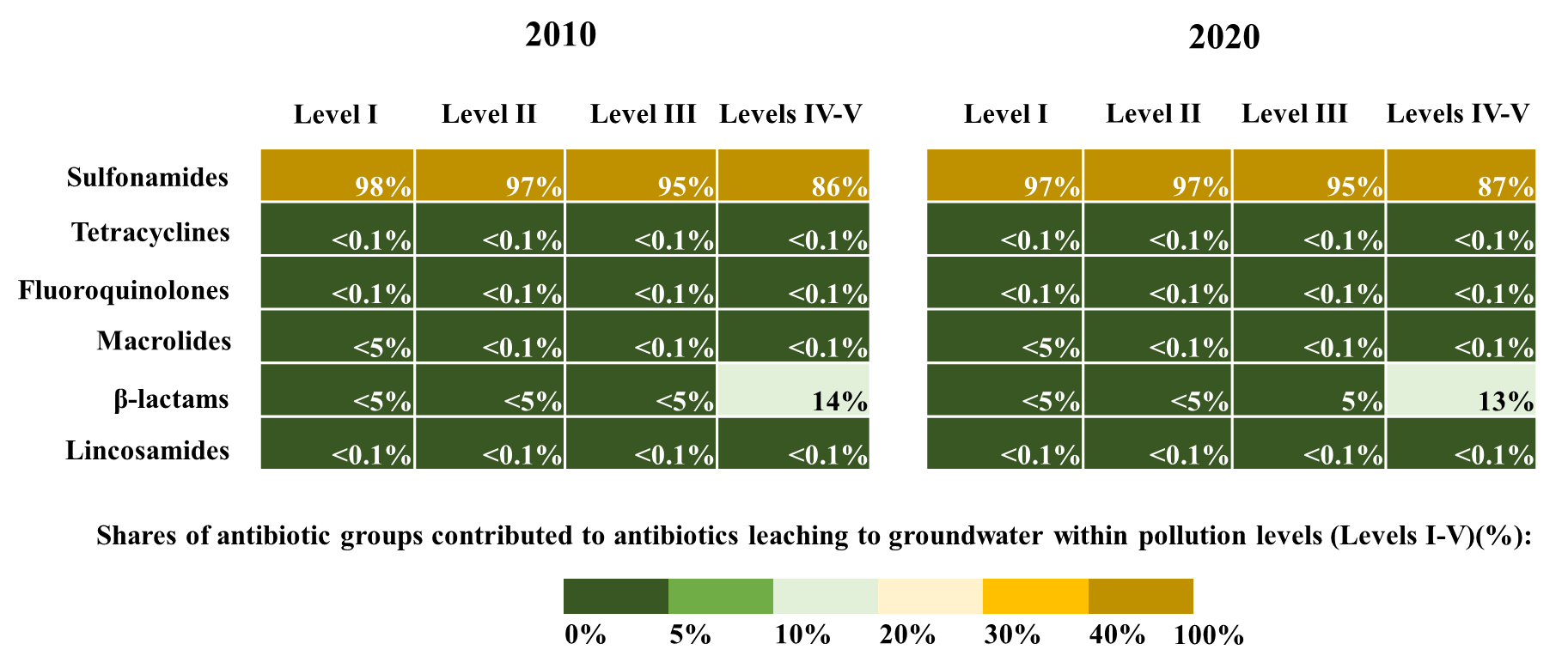
**

**Fig. S12. Shares of antibiotic groups in groundwater pollution in Levels I-V sub-basins (%).** Sources: the MARINA-Antibiotics model (see Section 2.1 for the model description in the main manuscript). Levels I-V refer to the pollution levels of total antibiotic leaching to groundwater (definition see Section 2.3 in the main manuscript).


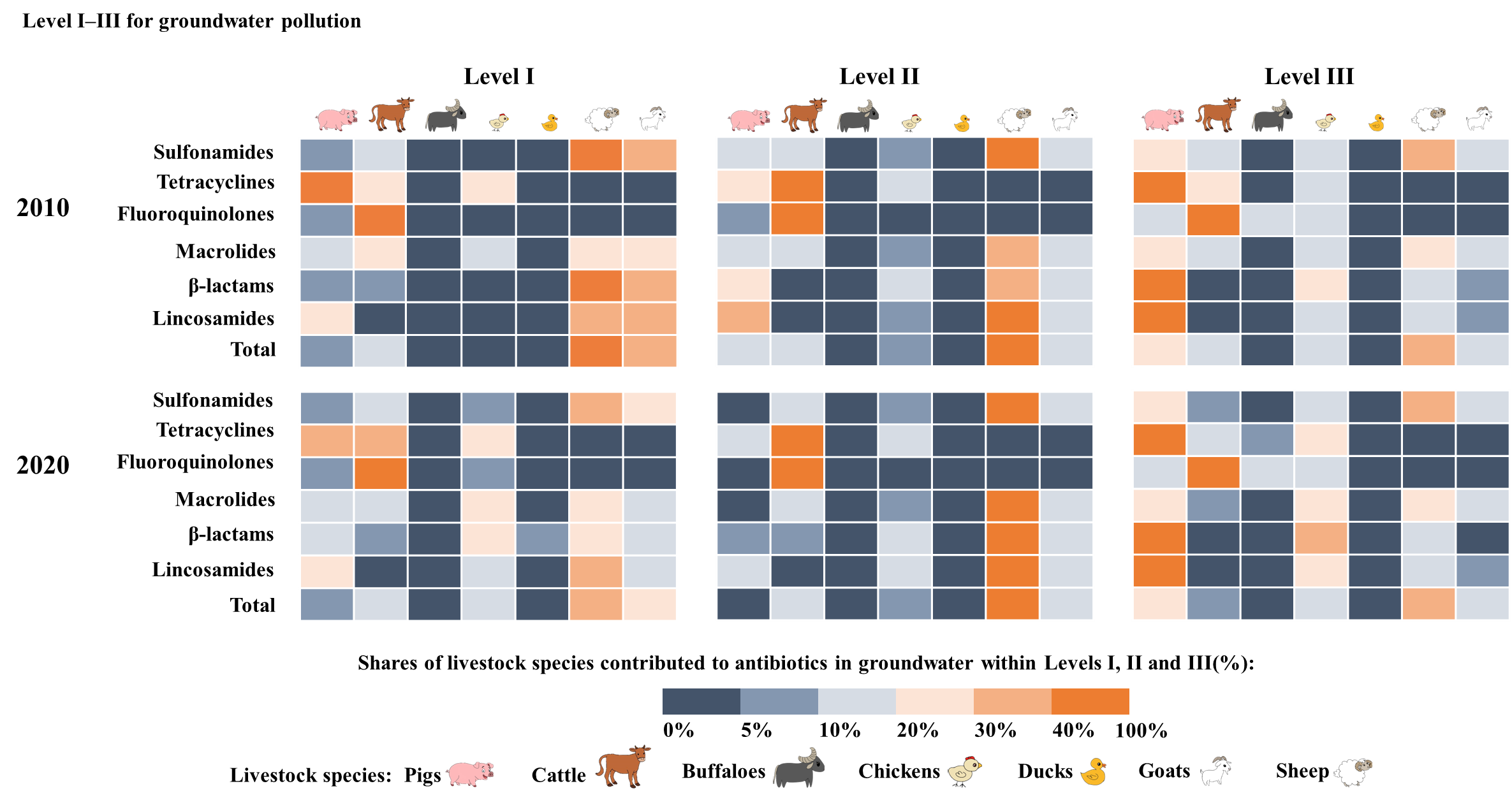


**Fig. S13. Shares of individual antibiotic groups in the total leaching into groundwater in sub-basins that belong to pollution levels I-III (%).** Sources: the MARINA-Antibiotics model (see Section 2.1 for the model description in the main manuscript). Levels I-III refer to the pollution levels of total antibiotic leaching into groundwater (definition see Section 2.3 in the main manuscript).

**Fig. S14. Model validation of soil organic carbon content input data for the MARINA-Antibiotics model.** We compare our model input data to HWSD v2 (Harmonized World Soils Database version 2) [5] for soil organic carbon content at the sub-basin scale (kg C/kg soil). R_P_ ^2^ and R_NSE_^2^ are Pearson’s coefficient of determination (fraction, 0-1) and the Nash-Sutcliffe efficiency (fraction, 0–1). Our comparisons indicate acceptable model performance, according to Moriasi, et al. [6]. The map shows the soil organic content per sub-basin (kg C/ kg soil) used in the MARINA-Antibiotics model. Sources: the MARINA-Antibiotics model (see Section 2.1 for the model description in the main manuscript).

**Fig. S15. Model validation of soil saturation input data for the MARINA-Antibiotics model.** We compare our model input data with GLEAM v3 model (Global Land Evaporation Amsterdam Model)[7, 8] for soil saturation at the sub-basin scale (cm^3^/cm^3^). The map shows the soil saturation per sub-basins (cm^3^/cm^3^). Data from the GLEAM v3 model are calculated from the long-term yearly averages. This long-term averages are calculated from the entire simulation for 43 years. R_P_ ^2^ and R_NSE_^2^ are Pearson’s coefficient of determination (fraction, 0-1) and the Nash-Sutcliffe efficiency (fraction, 0–1). Our comparisons indicate acceptable model performance, according to Moriasi, et al. [6]. Sources: the MARINA-Antibiotics model (see Section 2.1 for the model description in the main manuscript).

**Fig. S16. Model validation of soil pH input data for the MARINA-Antibiotics model.** We compare our model input data with the soil pH to Poggio, et al. [9]at the sub-basin scale (-). Map shows the spatial distribution of soil pH for sub-basins in China (-). R_P_ ^2^ and R_NSE_^2^ are Pearson’s coefficient of determination (fraction, 0-1) and the Nash-Sutcliffe efficiency (fraction, 0–1). Our comparisons indicate acceptable model performance according to Moriasi, et al. [6]. Sources: the MARINA-Antibiotics model (see Section 2.1 for the model description in the main manuscript).

**Fig. S17. Model validation of soil temperature input data for the MARINA-Antibiotics model.** We compare our model input data to Johan, et al. [10] for soil temperature at the sub-basin scale (K). Map shows the spatial distribution of soil temperature for sub-basins in China (K). R_P_ ^2^ and R_NSE_^2^ are Pearson’s coefficient of determination (fraction, 0-1) and the Nash-Sutcliffe efficiency (fraction, 0–1). Our comparisons indicate acceptable model performance according to Moriasi, et al. [6]. Sources: the MARINA-Antibiotics model (see Section 2.1 for the model description in the main manuscript).

**
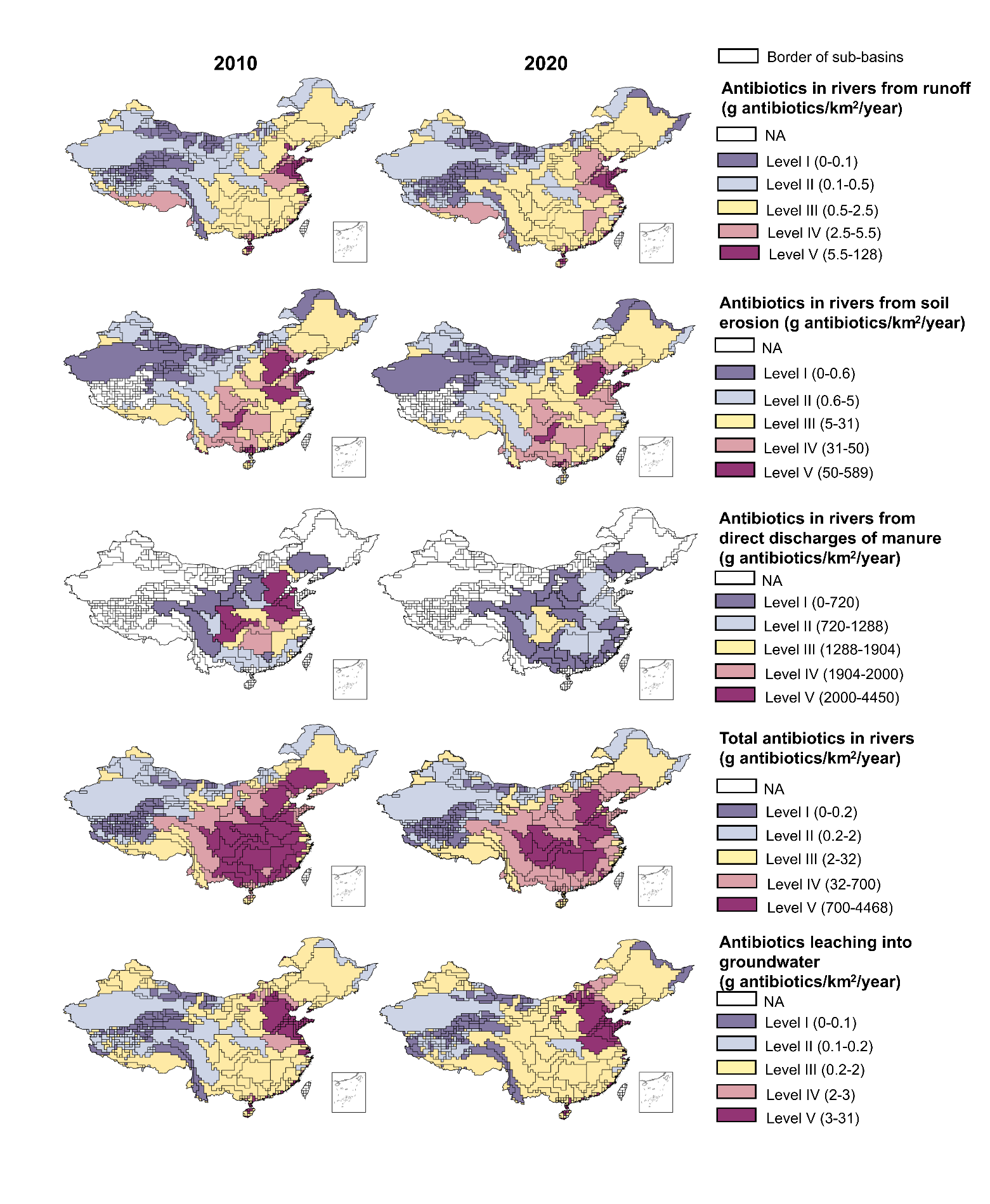
**

**Fig. S18. Antibiotic pollution in waters from livestock production at the sub-basin scale in China in 2010 and 2020 (g antibiotics/km^2^/year).** The maps show antibiotics entering rivers from surface runoff, soil erosion and direct discharges of livestock manure, as well as antibiotics entering groundwater from leaching (g/km^2^/year). These inputs are classified into five pollution levels from low (Level I) to high (Level V). Levels IV and V are considered pollution hotspots (definition see Section 2.3 in the main manuscript). Sources: the MARINA-Antibiotics (China-1.0) model (see Section 2.1 in the main manuscript for the model description). NA is short for No Data.

**Fig. S19. Changes in antibiotic inputs into rivers at sub-basin scale between 2010 and 2020 (%).** Sources: the MARINA-Antibiotics (China-1.0) model (see Section 2.1 for the model description in the main manuscript).

**Fig. S20. Changes in antibiotic leaching into groundwater at sub-basin scale between 2010 and 2020 (%).** Sources: the MARINA-Antibiotics (China-1.0) model (see Section 2.1 for the model description in the main manuscript).

**Table S1. Equations of the MARINA-Antibiotics model to quantify manure-associated inputs of antibiotics to rivers and groundwater from grazing and storage systems in 395 Chinese sub-basins.** Inputs of antibiotics are quantified from point and diffuse sources. Point sources include the direct discharges of manure to rivers without degradation during storage systems. Diffuse sources include inputs of antibiotics to rivers from land via runoff, and soil erosion, and leaching of antibiotics to groundwater. MARINA-Antibiotics is short for **M**odel to **A**ssess **R**iver Inputs of polluta**N**ts to se**A**s for Antibiotics in freshwater. Abbreviations are explained in Table S2.

| Equations | Numbers |
| --- | --- |
| $\boldsymbol{WSdif}_{\boldsymbol{A, i, j}}\boldsymbol{=}\boldsymbol{WSdif}_{\boldsymbol{A,}\boldsymbol{sg}\boldsymbol{, i, j}}\boldsymbol{+}\boldsymbol{WSdif}_{\boldsymbol{A,}\boldsymbol{ss}\boldsymbol{, i, j}}$ | **Eq. S1** |
| ${WSdif}_{A,sg,i,j}=X_{A,i,j}\times{fr}_{sg,i,j}$ | Eq. S2 |
| $X_{A,i,j}=H_{i,j}\times R_{A,i,j}$ | Eq. S3 |
| ${WSdif}_{A,i,ss,j}=S_{ss,A,i,j}\times\left( 1-{fr}_{d,i,j} \right)$ | Eq. S4 |
| $S_{ss,A,i, j}=X_{A,i,j}\times{fr}_{ss,i,j}\times\left( 1-{fr}_{ss,de,i,j} \right)$ | Eq. S5 |
| $\boldsymbol{RS}_{\boldsymbol{A, i, j}}\boldsymbol{=}\boldsymbol{RSdif}_{\boldsymbol{A,i,j}}\boldsymbol{+}\boldsymbol{RSpnt}_{\boldsymbol{A, i, j}}$ | **Eq. S6** |
| ${RSpnt}_{A, i, j}= S_{ss,A,i,j} \times{fr}_{d,i,j}$ | Eq. S7 |
| ${RSdif}_{A, i, j}= {RSsr}_{A,i,j}+{RSes}_{A,i,j}$ | Eq. S8 |
| ${RSsr}_{A,i,j}={Ssol}_{A,i,j}\times{FEsr}_{j}$ | Eq. S9 |
| ${RSes}_{A,i,j}={Spar}_{A,i,j}\times{FEes}_{j}$ | Eq. S10 |
| ${FEsr}_{j}=\frac{{Rnat}_{j}}{P_{j}}$ | Eq. S11 |
| ${Rnat}_{j}=$ ${Q_{nat,j}}/{{Area}_{j}}$ | Eq. S12 |
| ${Ssol}_{A,i,j}= {Ssol}_{ss, A,i,j}+{Ssol}_{sg,A,i,j}$ | Eq. S13 |
| ${Spar}_{A,i,j}={Spar}_{ss,A,i,j}+{Spar}_{sg,A,i,j}$ | Eq. S14 |
| ${Spar}_{ss,A,i,j}= {WSdif}_{A,i,ss,j}\times{FS}_{par,A,j}\times{FS}_{ss,de,A,j}$ | Eq. S15 |
| ${Spar}_{sg,A,i,j}= {WSdif}_{A,i,sg,j}\times{FS}_{par,A,j}\times{FS}_{sg,de,A,j}$ | Eq. S16 |
| ${Ssol}_{ss,A,i,j}={WSdif}_{A,i,ss,j}\times{FS}_{sol,A,j}\times{FS}_{ss,de,A,j}$ | Eq. S17 |
| ${Ssol}_{sg,A,i,j}={WSdif}_{A,i,sg,j}\times{FS}_{sol,A,j}\times{FS}_{sg,de,A,j}$ | Eq. S18 |
| ${FS}_{ss,de,A,j}=e^{-{kt}_{ss}}$ | Eq. S19 |
| ${FS}_{sg,de,A,j}=e^{-{kt}_{sg}}$ | Eq. S20 |
| $k\boldsymbol{=}{fr}_{bio,j}\times{fr}_{pH,j}\times\frac{ln2}{{DT}_{50}}\times\frac{{SOC}_{j}}{{SOC}_{ref}}$ | Eq. S21 |
| ${fr}_{pH,j}=\frac{C_{H^{+},j}}{C_{H^{+},j}+K_{pH,L}}\times\frac{K_{pH,U}}{C_{H^{+},j}+K_{pH,U}}$ | Eq. S22 |
| ${fr}_{bio,j}=min\left\{ {fr}_{T,j},{fr}_{S,j}/max\left( {fr}_{S,j} \right) \right\}$ | Eq. S23 |
| ${fr}_{T,j}=\left( \frac{e^{T,j}}{e^{T_{L}}+e^{T,j}} \right)^{0.15}\times\left( \frac{e^{T_{U}}}{e^{T_{U}}+e^{T,j}} \right)^{0.35}$ | Eq. S24 |
| ${fr}_{S,j}=\left( \frac{S_{j}}{S_{L}+S_{j}} \right)\times\left( \frac{S_{U}}{S_{U}+S_{j}} \right)$ | Eq. S25 |
| $\boldsymbol{GW}_{\boldsymbol{A,i, j}}\boldsymbol{=}\left( \boldsymbol{Ssol}_{\boldsymbol{A,i,j}}\boldsymbol{-}\boldsymbol{RSsr}_{\boldsymbol{A,i,j}} \right)\boldsymbol{\times}\boldsymbol{fr}_{\boldsymbol{le,A,j}}$ | **Eq. S26** |

**Table S2. Descriptions of the abbreviations in Table S1.**

| Parameters | Descriptions | Unit | Processing categories (Table S3 and Fig. S1) | Sources |
| --- | --- | --- | --- | --- |
| *i* | Livestock species: buffaloes, cattle, chickens, ducks, goats, pigs, sheep | **-** | **-** | **-** |
| *j* | Sub-basin | **-** | **-** | **-** |
| *A* | Antibiotics include six groups: Sulfonamides, Tetracyclines, Fluoroquinolones, Macrolides,β-lactams, and Lincosamides. | **-** | **-** | **-** |
| *sg* | Grazing system | **-** | **-** | **-** |
| *ss* | Storage system | **-** | **-** | **-** |
| *d* | Direct discharges of manure to rivers (without treatment) | **-** | **-** | **-** |
| *de* | Antibiotics in livestock manure that are degraded during manure storage (e.g. composting, anaerobic digestion, etc.) | **-** | **-** | **-** |
| ${WSdif}_{A, i, j}$ | The application of antibiotic group (*A*) to agricultural land from the manure of livestock species (*i*) in sub-basin (*j*). | kg antibiotics/year | **-** | Calculated. |
| ${WSdif}_{A,sg, i, j}$ | The application of antibiotic group (*A*) to agricultural land from the manure of livestock species (*i*) from grazing system (*sg*) in sub-basin (*j*). | kg antibiotics/year | **-** | Calculated. |
| ${WSdif}_{A,i,ss,j}$ | The application of antibiotic group (*A*) to agricultural land from the manure of livestock (*i*) from the storage system (*ss*) in sub-basin (*j*). | kg antibiotics/year | **-** | Calculated. |
| $X_{A,i,j}$ | Total excretion of antibiotics (*A*) in the manure of livestock species (*i*) in sub-basin (*j*) | kg antibiotics/year | **-** | Calculated. |
| $H_{i,j}$ | Number of livestock species (*i*) in sub-basin (*j*) | head/year | 1 | [1, 11-17] |
| $R_{A,i,j}$ | Excretion rates of antibiotics (*A*) in the manure of livestock species (*i*) in sub-basin (*j*) | kg antibiotics/head/year | 2 | Calculated based on values from literature [18-23]. |
| ${fr}_{d,i,j}$ | Fraction of direct discharges (*d*) of antibiotics’ manure to rivers from livestock species (*i*) in sub-basin (*j*) | 0-1 | 3 | [1] for 2010. For 2020, this fraction calculated based on Zhu, et al. [24] |
| ${fr}_{sg,i,j}$ | Fraction of grazing system (*sg*) for livestock species (*i*) in sub-basin (*j*) | 0-1 | 3 | [1]. |
| ${fr}_{ss,i,j}$ | Fraction of storage system (*ss*) for livestock species (*i*) in sub-basin (*j*) | 0-1 | 3 | [1]. |
| ${fr}_{ss,de,i,j}$ | Degradation efficiencies of antibiotics in the manure of livestock species (*i*) degraded in the storage system (*ss*) in sub-basin (*j*) | 0-1 | 2 | [25-29]. |
| $S_{ss,A,i,j}$ | Excretion of antibiotics in the manure of livestock species (*i*) in storage systems (*ss*) in sub-basin (*j*) | kg antibiotics/year | - | Calculated. |
| ${RS}_{A, i, j}$ | Total inputs of antibiotic group (*A*) to rivers (*RS*) from the manure of livestock species (*i*) in sub-basin (*j*) | kg antibiotics/year | - | Calculated. |
| ${RSdif}_{A,i,j}$ | Total inputs of antibiotic groups (*A*) to rivers (*RS*) from the manure of livestock species (*i*) from diffuse sources (*dif*) in sub-basin (*j*) | kg antibiotics/year | - | Calculated. |
| ${RSpnt}_{A,i, j}$ | Inputs of antibiotic groups (*A*) to rivers (*RS*) from the manure of livestock species (*i*) from point sources (*pnt*) in sub-basin (*j*) | kg antibiotics/year | - | Calculated. |
| ${RSsr}_{A,i,j}$ | Inputs of antibiotic group (*A*) to rivers from the manure of livestock species (*i*) via runoff (*sr*) in sub-basin (*j*) | kg antibiotics/year | - | Calculated. |
| ${RSes}_{A,i,j}$ | Inputs of antibiotic group (*A*) to rivers from the manure of livestock species (*i*) via soil erosion (*es*) in sub-basin (*j*) | kg antibiotics/year | - | Calculated. |
| ${Ssol}_{A,i,j}$ | The amount of antibiotic group (*A*) from the manure of livestock species (*i*) that is retained in the soil solution (*Ssol*) after sorption and degradation in sub-basin (*j*) | kg antibiotics/year | - | Calculated. |
| ${FEsr}_{j}$ | The export fraction (*FE*) of antibiotics that enter rivers from the soil solution via runoff (*sr*) in sub-basin (*j*) | 0-1 | - | Calculated. |
| ${Spar}_{A,i,j}$ | The total amount of antibiotic group (*A*) from the manure of livestock species (*i*) that is retained in the soil particles (*Spar*) after sorption and degradation in sub-basin (*j*) | kg antibiotics/year | - | Calculated. |
| ${FEes}_{j}$ | The export fraction (*FE*) of antibiotics that enter rivers from the soil particles via soil erosion (*es*) in sub-basin (*j*). | 0-1 | 2 | Calculated based on Liu, et al. [30]. |
| ${Rnat}_{j}$ | Annual surface runoff from land to streams in sub-basin (j) | meter | - | Calculated. |
| $P_{j}$ | 30-year (1980-2010 and 1990-2020) average annual precipitation in sub-basin (j) | mm | 5 | Calculated based on Weedon, et al. [31] and Stefan and Matthias [32]. |
| $Q_{nat,j}$ | Natural (nat) river discharge at the outlet of sub-basin (j) before water consumption | km^3^/year | 4 | Calculated based on Weedon, et al. [31] and Stefan and Matthias [32]. |
| ${Area}_{j}$ | The drainage area of sub-basin (j) | km^2^ | 4 | [33]. |
| $S$ | Soil texture in sub-basin (j) | - | 10 | [34]. |
| ${Ssol}_{ss,A,i,j}$ | The amount of antibiotic group (*A*) from the manure of livestock species (*i*) that is retained in the soil solution (*Ssol*) from the storage systems (*ss*) after sorption and degradation in the sub-basin (*j*) | kg antibiotics/year | - | Calculated. |
| ${Ssol}_{sg,A,i,j}$ | The amount of antibiotic group (*A*) that is retained in the soil solution (*Ssol*) from the manure of livestock species (*i*) from the grazing systems (*sg*) after sorption and degradation in the sub-basin (*j*) | kg antibiotics/year | - | Calculated. |
| ${Spar}_{ss,A,i,j}$ | The total amount of antibiotic group (*A*) that is retained in the soil particle (*Spar*) from the manure of livestock species (*i*) from the storage systems (*ss*) after sorption and degradation in the sub-basin (*j*) | kg antibiotics/year | - | Calculated. |
| ${Spar}_{sg,A,i,j}$ | The total amount of antibiotic group (*A*) that is retained in the soil particle (*Spar*) from the manure of livestock species (*i*) from the grazing systems (*sg*) after sorption and degradation in the sub-basin (*j*). | kg antibiotics/year | - | Calculated. |
| ${FS}_{par,A,j}$ | The sorption fractions (*FS*) of antibiotics (*A*) in the soil particle (*par*) in sub-basin (*j*) | 0-1 | 2 | Calculated based on K_d_ values and the maximum water-holding capacity [35-46]. |
| ${FS}_{sol,A,j}$ | The sorption fractions (*FS*) of antibiotics (*A*) in the soil solution (*sol*) in sub-basin (*j*) | 0-1 | 2 | Calculated based on K_d_ values and the maximum water-holding capacity [35-46]. |
| ${FS}_{ss,de,A,j}$ | The degradation fraction (*FS*) of antibiotics (*A*) in the soil after the manure application from the storage systems (*ss*) in sub-basin (*j*) | 0-1 | - | Calculated. |
| ${FS}_{sg,de,A,j}$ | The degradation fraction (*FS*) of antibiotics (*A*) in the soil after the manure application from the grazing systems (*sg*) in sub-basin (*j*) | 0-1 | - | Calculated. |
| $t_{ss,j}$ | The degradation duration in the soil after the application of livestock manure (*i*) from the storage systems (*ss*) in sub-basin (*j*) | Days | 2 | Calculated based on MARA [47]. |
| $t_{sg,j}$ | The degradation duration in the soil after the application of livestock manure (*i*) from the grazing systems (*sg*) in sub-basin (*j*) | Days | 2 | Assumed based on Wöhler, et al. [48], MOA [49], and MOA [50]. |
| $k_{ss,j}$ | The degradation rate (*k*) of antibiotics in the soil after the application of livestock manure from the storage systems (*ss*) in sub-basin (*j*). This degradation rate occurs by first-order kinetics and considers soil texture, biological activities, soil moisture content, soil temperature, pH, and organic carbon content in the soil | kg antibiotics/day | - | Calculated. |
| $k_{sg,j}$ | The degradation rate (*k*) of antibiotics in soil after the application of livestock manure from the grazing systems (*sg*) in sub-basin (*j*). This degradation rate occurs by first-order kinetics and considers soil texture, biological activity, soil moisture content, soil temperature, pH, and organic carbon content in the soil | kg antibiotics/day | - | Calculated. |
| ${fr}_{bio,j}$ | Fraction (*fr*) of the biological response (*bio*) to the changes induced by soil saturation and temperature in sub-basin (*j*) | 0-1 | - | Calculated. |
| ${fr}_{pH,j}$ | Fraction (*fr*) of the effect of pH (*pH*) on antibiotic degradation in the soil in sub-basin (*j*) | 0-1 | - | Calculated. |
| ${SOC}_{j}$ | Soil organic carbon content (*SOC*) in the soil in sub-basin (*j*) | kg C/kg soil | 7 | Calculated based on Poggio, et al. [9]. |
| ${SOC}_{ref}$ | The median of soil organic carbon content (*SOC*) among all the sub-basins in China at rootzone (≤60 cm) | kg C/kg soil | 7 | ${SOC}_{ref}$ is the global median of soil organic carbon content (*SOC*) among all the sub-basins. |
| ${DT}_{50}$ | The half-life of antibiotics in different soil textures in sub-basin (*j*) | Days | 2 | [18, 35, 39-41, 51-55]. |
| $C_{H^{+},j}$ | The concentration of H^+^ in the soil that is calculated based on the pH value in sub-basin (*j*) | mol/L | 7 | Calculated based on Poggio, et al. [9]. |
| $K_{pH,L}$ | The concentration of H^+^ for the lower (*L*) inhibition around the optimal value of pH 7. This concentration is 1$\times$10^-9^ | mol/L | 2 | [56]. |
| $K_{pH,U}$ | The concentration of H^+^ for the upper (*U*) inhibition around the optimal value of pH 7. This concentration is 1$\times$10^-5^ | mol/L | 2 | [56]. |
| ${fr}_{T,j}$ | Fraction (*fr*) of the biological response to the changes induced by soil temperature (*T*) in sub-basin (*j*) | 0-1 | - | Calculated. |
| $T_{j}$ | Soil temperature (*T*) in sub-basin (*j*) | K | 6 | [57]. |
| $T_{L}$ | The lower (*L*) soil response temperatures (*T*) for mesophiles give the optimal response. This lower response temperature is 288.15 K. | K | 2 | [56]. |
| $T_{U}$ | The upper (*U*) soil response temperatures (*T*) for mesophiles give the optimal response. This upper response temperature is 313.15 K. | K | 2 | [56]. |
| $S_{j}$ | Soil saturated water content (*S*) in sub-basin (*j*) | cm^3^/cm^3^ | 8 | [58]. |
| $S_{L}$ and $S_{U}$ | Soil saturated water (*S*) for an optimal biological response. $S_{L}=$ $S_{U}$=0.46 | cm^3^/cm^3^ | 2 | [56]. |
| ${GW}_{A, i, j}$ | Total inputs of antibiotic group (*A*) that are leached to groundwater (*GW*) from the manure application on agricultural land from livestock species (*i*) in sub-basin (*j*) | kg antibiotics/year | - | Calculated. |
| ${fr}_{le,A,j}$ | The potential leaching fraction (${fr}_{le}$) of antibiotic group (*A*) in the soil solution below 200 cm of the soil in sub-basin (*j*) | 0-1 | 2 | Calculated based on K_d_ values and the maximum water-holding capacity [35-46] |

**Table A.3. Descriptions of how model inputs are processed to sub-basins for the MARINA-Antibiotics model** (Model to Assess River Inputs of pollutaNts to seAs for Antibiotics in freshwater). The model sources and equations are described in Fig. S2 and Table S1.

| Model input category from Fig. S2 | Equations in Table S1 | Descriptions of how model inputs are processed to sub-basins |
| --- | --- | --- |
| 1 | Eq. S3 | **Livestock numbers (**$\boldsymbol{H}_{\boldsymbol{i,j}}$**, head/year)** are provided by Vermeulen, et al. [2] for the year 2010. These data are collected by Vermeulen, et al. [2] from the Gridded Livestock of World v3.0 dataset and FAO datasets for 11 livestock species at the country level. These livestock numbers are used in the Global Waterborne Pathogen model for *Cryptosporidium* from Livestock (GloWpa-Crypto-L1) [2]. For the year 2020, we calculated livestock number based on Chinese statisitc yearbook and the Gridded Livstock of World v4.0 dataset [11-17] for seven livestock species.  In our study, we used the sum function of Zonal Statistics in ArcGIS to aggregate livestock numbers from 0.5˚ $\times$ 0.5˚ grids to 395 sub-basins in China. We aggregated livestock numbers to sub-basins by summing them over the corresponding grids of 0.5˚ based on the method of Li, et al. [1] for the years 2010 and 2020. |
| 2 | Eqs. S3, S5, S10, and S15-S23 | Excretion rates of antibiotics by livestock species **(**$\boldsymbol{R}_{\boldsymbol{A,i,j}}$**, kg antibiotics/head/year)** were derived from existing studies [18-23]. Excretion rates of antibiotics for pigs and chickens were province-specific [18]. We aggregated provincial values to sub-basins as follows: we assigned provincial values (kg of antibiotics/head/year) to 0.5˚ $\times$ 0.5˚ grids. Then, we multiplied gridded excretion rates (kg of antibiotics/head/year) with the livestock number (head/year) to get the total excretion per grid (kg of antibiotics/year). After that, we used the sum function of Zonal Statistics in ArcGIS to sum the total excretion for sub-basins over the corresponding grids to get the total excretion per sub-basin (kg of antibiotics/year). Finally, we divided the total excretion per sub-basin (kg of antibiotics/ year) by the total number of livestock species per sub-basin (head/year) to get excretion rates (kg of antibiotics/ head/year) in the sub-basins.  Excretion rates of antibiotics for cattle, sheep, and goats **(**$\boldsymbol{R}_{\boldsymbol{A,i,j}}$**, kg antibiotics/head/year)** were calculated based on the concentration of antibiotics in livestock manure (kg of antibiotics/kg manure) [19-23] and the manure production amount per livestock species (kg manure/head/year) [59]. The concentration of antibiotics in livestock manure (kg antibiotics/kg manure) was presented at the provincial level. First, we used the concentration of antibiotics in livestock manure (kg antibiotics/kg manure) and multiplied it with the manure production amount per livestock species (kg manure /head/year) to get the excretion rates of antibiotics for cattle, sheep, and goats at the provincial level (kg antibiotics/head/year). Then, we followed the same steps for the pigs and chickens above to aggregate provincial data to sub-basins. The excretion rates of antibiotics for buffaloes were not available in the existing studies. We assumed the same as for cattle. In our study, we considered 24 antibiotics. Most of the excretion rates of antibiotics for duck were derived from Zhou, et al. [19] at the provincial level. The rest of the excretion rates of antibiotics (e.g., Sulfameter, Tylosin, Roxithromycin, Penicillin, and Lincomycin) for ducks were not available in the existing studies. We assumed the excretion rates of antibiotics (e.g., Sulfameter, Tylosin, Roxithromycin, Penicillin, and Lincomycin) for ducks in China were the same as the excretion rates for chickens at the provincial level. Then, we followed the same steps for the chickens above to aggregate provincial data to sub-basins of the excretion rates for ducks in the sub-basins.  Degradation efficiencies of antibiotics in the manure of livestock species degraded in the storage systems **(**$\boldsymbol{fr}_{\boldsymbol{ss,de,i,j}}$**, 0-1).** This parameter was derived from Bohrer, et al. [25], Chu, et al. [26], Gaballah, et al. [27], Han, et al. [28], Spielmeyer [29] and was livestock- and antibiotic-specific. We used the averaged value of the degradation efficiencies of antibiotics in livestock manure from the literature to estimate the situation in the years 2010 and 2020. This is because the literature provided data on antibiotic degradation efficiency in storage systems from 2010 to 2020.  Antibiotic half-life in the soil **(**$\boldsymbol{DT}_{\mathbf{50}}$**, days)**. This was derived from literature using the antibiotic half-life that is the soil texture- and antibiotic-specific [18, 35, 39-41, 51-55]. Then, we obtained the half-lives of 24 antibiotics in six soil textures. After that, we averaged these values over antibiotic groups and soil textures for each sub-basin.  $\boldsymbol{FS}_{\boldsymbol{par,A,j}}$ and $\boldsymbol{FS}_{\boldsymbol{sol,A,j}}$ are the sorption fractions (*FS*) of antibiotics (A) in the soil particle (*par*) and solution (*sol*) in sub-basin (*j*) (0–1). These fractions of antibiotics are calculated following the approach of Pan and Chu [35]. Pan and Chu [35] analyzed the adsorption of antibiotics in clay loam agricultural soil in China using the K_d_ value (the linear adsorption constant (kg/kg)) and maximum water-holding capacity. In our study, we followed this method to calculate sorption fractions and considered the equilibrium between soil particle and solution. The calculation of ${FS}_{par,A,j}$ and ${FS}_{sol,A,j}$ was done in three steps. **First,** we collected the K_d_ values (the linear adsorption constant (kg/kg)) by antibiotic groups and soil textures from the available literature [35-45]. Then, we statistically averaged the K_d_ values over antibiotic groups and soil textures for each sub-basin. **Second,** we prepared the maximum water-holding capacity (0-1) for each soil texture from Pan and Chu [35] and Geohring, et al. [46]. After that, we assigned the maximum water-holding capacity to sub-basins based on their soil texture. **Third,** the fraction of antibiotics in the soil particle in the sub-basin was done as follows: ${FS}_{par,A,j}$ = (the K_d_ value (kg/kg)$\times$maximum water-holding capacity (0-1))$\div$(the K_d_ value (kg/kg) + maximum water-holding capacity (0-1)). The fraction of antibiotics in the soil solution in sub-basins was done as follow: ${FS}_{sol,A,j}$= ${FS}_{par,A,j}\div$the K_d_ value (kg/kg).  $t_{j}$ was the degradation duration in the soil after the application of livestock manure from the manure management systems in sub-basins (*j*) (day). This parameter was different for the manure application from storage and grazing systems. For the manure application from storage systems, we estimated the degradation duration in sub-basins based on the crop calendar [47]. This calendar showed the planting months for the crops in eight regions across China. First, we identified the planting months at the provincial level in each region. Second, we assumed that manure was applied during the months of planting crops. Third, we summed the manure application times (1-3 times) per province within the year. Fourth, we assigned the data on the times of manure applications per province on the sub-basins. Then, we determined the majority manure application times per sub-basin by using the majority function of Zonal Statistics in ArcGIS. For the grazing systems, we assumed that livestock species graze for half a year based on Wöhler, et al. [48], MOA [49], and MOA [50]. This implies that manure is applied on agricultural land during those times within the year.  Several model inputs were derived from the PEST-CHEMGRIDS v1.0 model [60]. These data were soil response temperature for mesophiles: $T_{L}$ and $T_{U}$, the concentration of H^+^ for the inhibition around the optimal value of pH 7: $K_{pH,L}$ and $K_{pH,U}$, and soil-saturated water for an optimal biological response: $S_{L}$ and $S_{U}$. In our study, we used the same value as they applied in the PES-CHEMGRIDS v 1.0 model for all sub-basins in China. |
| 3 and 4 | Eqs. S2, S7 and S12 | Several model inputs were derived from the MARINA-Global model (Fig. S2) [1]. These model inputs were sub-basin-specific, such as the drainage area of sub-basins ($\boldsymbol{Area}_{\boldsymbol{j}}$, km^2^), natural river discharge ($\boldsymbol{Q}_{\boldsymbol{nat,j}}$, km^3^/year), and the size of the sub-basins. Model inputs for the drainage area and natural river discharge at the sub-basin outlets were derived directly from the MARINA-Global model of Li, et al. [1], Strokal, et al. [61]. Some model inputs were livestock-specific including the fraction of direct discharge ($\boldsymbol{fr}_{\boldsymbol{d,i,j}}$, 0-1) of livestock manure to rivers and the fraction of grazing and storage systems for livestock species ($\boldsymbol{fr}_{\boldsymbol{sg,i,j}}$ and $\boldsymbol{fr}_{\boldsymbol{ss,i,j}}$, 0-1). This data was directly taken from the MARINA-Global model [1] for the year 2010. Our study area included 395 sub-basins. These sub-basins were delineated in earlier studies for the MARINA-Global model [1, 61] using the ddm 30 flow directions. The size of the sub-basin differed depending on the drainage areas of the river flows and their directions. The drainage area of the large river basins was divided into sub-basins depending on their hydrological characteristics such as the main channel, tributaries, etc. In the MARINA-Global model, the sub-basins were classified into upper-, middle-, and downstream. For the year 2020, we updated the fraction of direct discharges of livestock manure based on Zhu, et al. [24] |
| 5 | Eqs. S11 and S12 | ${FEsr}_{j}$ was the export fraction (*FE*) of antibiotics that enter rivers from the soil solution via runoff (*sr*) in sub-basin (*j*) (0-1). This fraction was calculated following the approach of Li, et al. [33]. This fraction was applied to the amount of antibiotics that is retained in the soil solution, which has the potential to enter rivers from runoff while accounting for degradation and sorption.  First, we derived the 30-year annual precipitation data from the WATCH Forcing Data methodology applied to ERA-Interim data (WFDEI) for the year 1980 to 2010 [31] for the year 2010. The VIC (Varibale Infiltration Capacity) hydrological model provided data for the period up to 2020. We calculated precipitation and natural river discharges for 2020 by averaging the data over the 30 years of the period 1990-2020 [32]. Then, we calculated the average annual precipitation per sub-basin (mm/year) by statistically averaging 30-year annual precipitation over 0.5˚ $\times$ 0.5˚ grids of the corresponding sub-basins using the average function of Zonal Statistics in ArcGIS. The output of this step was the average annual precipitation per sub-basin (mm/year) within 30 years (1980–2010 and 1990-2020). After that, we prepared the 30-year annual runoff per sub-basin. This was calculated based on the 30-year average annual natural river discharge in the sub-basin (km^3^/year) divided by their drainage area (km^2^), following the definition of Mayorga, et al. [62] and Strokal, et al. [63]. Then, we used the 30-year average annual runoff (km/year) to multiply it by 10^6^ to convert its unit from km/year to mm/year. Finally, the export fraction of antibiotics that is retained in the soil solution via runoff to rivers is calculated by dividing the average annual runoff (mm/year) divided by the average annual precipitation (mm/year) for each sub-basin. |
| 6 | Eq. S22 | $T_{j}$ was the soil temperature (T) in sub-basin (j) (K). This was derived from the soil temperature data from the CMADS-ST V1.0 (The China Meteorological Assimilation Driving Datasets for the SWAT model) [64]. This dataset quantified soil temperature in East Asia at 10 soil depths in 1/3˚ $\times$ 1/3 ˚ degree grids. Then, we calculated the average soil temperature at 60 cm of soil depth per sub-basin (T, K) by statistically averaging the soil temperature over 1/3˚ $\times$ 1/3 ˚ grids at 60 cm of soil depth for the corresponding sub-basins using the average function of Zonal Statistics in ArcGIS. The output of this step was the averaged soil temperature at 60 cm of soil depth per sub-basin (T, K). |
| 7 | Eqs. S19 and S20 | Soil organic carbon content in the soil in sub-basin (j) **(**$\boldsymbol{SOC}_{\boldsymbol{j}}$**, kg C/kg soil)** and the concentration of soil pH in water in sub-basin (j) **(**$\boldsymbol{C}_{\boldsymbol{H}^{\boldsymbol{+}}\boldsymbol{,j}}$**, mol/L).** First, we collected the soil organic carbon content (kg C/kg soil) and soil pH in H_2_O in soil (unitless) from the SoilGrids 2.0 at the resolution of 1 km$\times$1 km grids [9]. Then, we aggregated grided soil organic carbon content (kg C/kg soil) and soil pH in water (unitless) to sub-basins using ArcGIS. For soil organic carbon content (kg C/kg soil), we aggregated the grided data to the sub-basins by following the three steps below.  First, we prepared raster maps with the total area at each 0.5˚ $\times$ 0.5 ˚ grid (km^2^). We also collected the bulk density data (kg soil/km^3^) from SoilGrids 2.0 at a resolution of 1 km$\times$1 km grids [9]. We used the resample function in the ArcGIS to change the cell size of soil organic carbon content (kg C/kg soil) and bulk density data (kg soil/ km^3^) from 1 km$\times$1 km grids to 0.5˚ $\times$ 0.5 ˚ grids. Then, we multiplied the gridded soil organic carbon content (kg C/ kg soil) with bulk density data (kg soil per km^3^), total area (km^2^), and soil depth (km) to get the total soil organic carbon per grid (kg C). After that, we used the sum function of Zonal Statistics in ArcGIS to sum the total soil organic carbon for sub-basins over corresponding grids to get the total soil organic carbon per sub-basin (kg C).  Second, we calculated the bulk density per sub-basin (kg soil /km^3^). We used the 0.5˚ $\times$ 0.5 ˚ grids of the bulk density to multiply with the total area of the grid (km^2^) and soil depth (km) to get the total bulk density per grid (kg soil). After that, we summed the total bulk density for sub-basins over corresponding grids to get the total bulk density per sub-basin (kg soil). This was done by the sum function of Zonal Statistics in ArcGIS.  Third, we calculated the soil organic carbon content in each sub-basin (kg C/kg soil). This was done by dividing the total soil organic carbon per sub-basin (kg C) (the result of the first step) by the total bulk density per sub-basin (the result of the second step). As a result, we obtained the soil organic carbon content for each sub-basin. ${SOC}_{reference}$ was the national median of the organic carbon content of the soil over the sub-basins. |
| 8 | Eq. S23 | Soil saturated water content in the sub-basin (j) **(**$\boldsymbol{S}_{\boldsymbol{j}}$**, cm^3^ /cm^3^).** The mean values of saturated water content were derived from the Kosgui model at a 1 km resolution (cm^3^ /cm^3^) [58]. We used the resample function in ArcGIS to change the cell size of saturated water content from 1 km$\times$1 km grids to 0.5˚ $\times$ 0.5 ˚ grids. We first convert the unit cm^3^/cm^3^ to km^3^/km^3^ by multiplying it by 1. Then, we multiplied the resampled saturated water content (km^3^ /km^3^) by the total area of the grid (km^2^) and soil depth (km) to get the total saturated water content per grid (km^3^). After that, we used the sum function of Zonal Statistics in ArcGIS to sum the saturated water content for sub-basins over corresponding grids to get the total saturated water content per sub-basin (km^3^). Finally, we divided the total saturated water content per sub-basin (km^3^) by the total area per sub-basin (km^2^) and soil depth (km). After that, we obtained the soil-saturated water content for each sub-basin. |
| 9 | Eqs. S13-S19 | Soil texture in sub-basin (j) ($\boldsymbol{S}$, unitless). Soil textures were obtained from the Nation Earth System Science Data Center (NESSDC) at a 1 km$\times$1 km grids resolution [34]. We determined the majority soil texture per sub-basin by using the majority function of Zonal Statistics in ArcGIS over corresponding grids of each sub-basin. |

**Table S4.** **Livestock unit coefficients in China that are derived from Eurostat [65]**. These coefficients are used to convert from livestock numbers to livestock number units for comparison purposes.

| **Livestock species** | **Livestock unit coefficient** |
| --- | --- |
| Cattle | 1 |
| Buffalo | 0.8 |
| Pig | 0.3 |
| Chicken | 0.01 (averaged value of broilers and laying hens) |
| Sheep | 0.1 |
| Goat | 0.1 |
| Duck | 0.01 |

**Table S5. Antibiotics for use in human medicine and food livestock production.** Source: Zhang, et al. [18], Huang, et al. [66]

| Groups | Antibiotics | Target groups |
| --- | --- | --- |
| Sulfonamides | Sulfachlorpyridazine | Livestock |
|  | Sulfadiazine | Human and Livestock |
|  | Sulfaguanidine | Human and Livestock |
|  | Sulfamonomethoxine | Human and Livestock |
|  | Sulfameter | Human and Livestock |
|  | Sulfamethazine | Human and Livestock |
|  | Sulfamethoxazole | Human and Livestock |
|  | Sulfathiazole | Human and Livestock |
|  | Sulfaquinoxaline | Livestock |
| Tetracyclines | Oxytetracycline | Human and Livestock |
|  | Tetracycline | Human and Livestock |
|  | Chlortetracycline | Human and Livestock |
|  | Doxycycline | Human and Livestock |
| Fluoroquinolones | Norfloxacin | Human and Livestock |
|  | Ciprofloxacin | Human and Livestock |
|  | Ofloxacin | Human and Livestock |
|  | Enrofloxacin | Human and Livestock |
|  | Fleroxacin | Human and Livestock |
|  | Pefloxacin | Human and Livestock |
|  | Difloxacin | Human and Livestock |
| Macrolides | Roxithromycin | Human and Livestock |
|  | Tylosin | Livestock |
| β-lactams | Penicillin | Human and Livestock |
| Lincosamides | Lincomycin | Human and Livestock |

**Table S6. Antibiotics in rivers resulted from diffuse sources (surface runoff and soil erosion) in northern and southern sub-basins in China between 2010 and 2020 (tonnes/year).**

|  | 2010 | 2020 | Changes among sub-basins (%) |
| --- | --- | --- | --- |
| Northern sub-basins | 20 | 23 | -56% to 206% |
| Southern sub-basins | 197 | 182 | -90% to 96% |
| Total | 217 | 205 | -6% |

**Table S7. Antibiotics in rivers resulted from point sources in northern and southern sub-basins in China between 2010 and 2020 (tonnes/year).**

|  | 2010 | 2020 | Changes among sub-basins (%) |
| --- | --- | --- | --- |
| Northern sub-basins | 263 | 125 | -58% to -48% |
| Southern sub-basins | 7874 | 3094 | -76% to -44% |
| Total | 8137 | 3219 | -60% |

**Table S8. Antibiotics leaching to groundwater in northern and southern sub-basins in China between 2010 and 2020 (tonnes/year).**

|  | 2010 | 2020 | Changes among sub-basins (%) |
| --- | --- | --- | --- |
| Northern sub-basins | 2 | 3 | -50% to 161% |
| Southern sub-basins | 8 | 8 | -90% to 110% |
| Total | 10 | 12 | 15% |

**Table S9. Antibiotics in livestock manure applied on land in northern and southern sub-basins in China between 2010 and 2020 (tonnes/year).**

|  | 2010 | 2020 | Changes among sub-basins (%) |
| --- | --- | --- | --- |
| Northern sub-basins | 1587 | 2183 | -62% to 311% |
| Southern sub-basins | 12429 | 11565 | -90% to 99% |
| Total | 14016 | 13748 | -2% |

**Table S10. Range of model inputs for antibiotic inputs into rivers and leaching into groundwater (0-1).** The model sources and equations are described in Fig. S2, Tables S1. and S2. Mean values and ranges exclude sub-basins with no data.

| Model inputs | 2010 | | 2020 | |
| --- | --- | --- | --- | --- |
|  | Mean | Ranges | Mean | Ranges |
| Application rate of antibiotic from livestock manure (kg antibiotics/km^2^/year) | 0.94 | 0.0004-18.02 | 0.92 | 0.006-7.8 |
| Export fraction of antibiotics resulting from surface runoff (0-1) | 0.32 | 0.016-0.88 | 0.32 | 0.015-0.86 |
| Export fraction of antibiotics resulting from soil erosion (0-1) | 0.13 | 0.01-0.64 | 0.13 | 0.01-0.64 |
| Fraction of direct discharges of livestock manure (0-1) | 0.61 | 0.31-0.77 | 0.27 | 0.11-0.49 |
| Leaching fraction (0-1) | 0.07 | 1.8*10^-6^-0.83 | 0.07 | 1.8*10^-6^-0.83 |

**REFERENCES**

[1] Y. Li, M. Wang, X. Chen, S. Cui, N. Hofstra, C. Kroeze, L. Ma, W. Xu, Q. Zhang, F. Zhang and M. Strokal. Multi-pollutant assessment of river pollution from livestock production worldwide. Water Research 209 (2022) 117906.

[2] L. C. Vermeulen, J. Benders, G. Medema and N. Hofstra. Global Cryptosporidium Loads from Livestock Manure. Environ Sci Technol 51(15) (2017) 8663-8671.

[3] NBSC, China Statistic Yearbook. In N. B. o. S. o. China, Ed. China Statistic Press: Beijing (2010).

[4] NBSC, China Statistic Yearbook. In N. B. o. S. o. China, Ed. China Statistic Press: Beijing (2020).

[5] F. Nachtergaele, H. van Velthuizen, L. Verelst, D. Wiberg, M. Henry, F. Chiozza, Y. Yigini, E. Aksoy, N. Batjes and E. Boateng, Harmonized World Soil Database version 2.0, Food and Agriculture Organization of the United Nations2023.

[6] D. N. Moriasi, J. G. Arnold, M. W. Van Liew, R. L. Bingner, R. D. Harmel and T. L. Veith. Model evaluation guidelines for systematic quantification of accuracy in watershed simulations. Transactions of the ASABE 50(3) (2007) 885-900.

[7] D. G. Miralles, T. R. H. Holmes, R. A. M. De Jeu, J. H. Gash, A. G. C. A. Meesters and A. J. Dolman. Global land-surface evaporation estimated from satellite-based observations. Hydrol. Earth Syst. Sci. 15(2) (2011) 453-469.

[8] B. Martens, D. G. Miralles, H. Lievens, R. Van Der Schalie, R. A. De Jeu, D. Fernández-Prieto, H. E. Beck, W. A. Dorigo and N. E. Verhoest. GLEAM v3: Satellite-based land evaporation and root-zone soil moisture. Geoscientific Model Development 10(5) (2017) 1903-1925.

[9] L. Poggio, L. M. de Sousa, N. H. Batjes, G. B. M. Heuvelink, B. Kempen, E. Ribeiro and D. Rossiter. SoilGrids 2.0: producing soil information for the globe with quantified spatial uncertainty. SOIL 7(1) (2021) 217-240.

[10] v. d. H. Johan, J. L. Jonas, A. Juha, B. A. Michael, D. F. Pieter, K. Julia, K. Martin, L. Miska, M. D. M. Ilya, C. Thomas, J. B. Joseph, H. Stef, H. K. David, N. Pekka, R. S. Brett, V. M. Koenraad, S. Consortium, N. Ivan and L. Jonathan, Global Soil Temperature code and data (Version 1) In Zenodo (2021).

[11] M. Gilbert, G. Cinardi, D. Da Re, W. G. R. Wint, D. Wisser and T. P. Robinson, Global sheep distribution in 2015 (5 minutes of arc). In V1 ed.; Harvard Dataverse: (2022).

[12] M. Gilbert, G. Cinardi, D. Da Re, W. G. R. Wint, D. Wisser and T. P. Robinson, Global chickens distribution in 2015 (5 minutes of arc). In V1 ed.; Harvard Dataverse: (2022).

[13] M. Gilbert, G. Cinardi, D. Da Re, W. G. R. Wint, D. Wisser and T. P. Robinson, Global pigs distribution in 2015 (5 minutes of arc). In V1 ed.; Harvard Dataverse: (2022).

[14] M. Gilbert, G. Cinardi, D. Da Re, W. G. R. Wint, D. Wisser and T. P. Robinson, Global goats distribution in 2015 (5 minutes of arc). In V1 ed.; Harvard Dataverse: (2022).

[15] M. Gilbert, G. Cinardi, D. Da Re, W. G. R. Wint, D. Wisser and T. P. Robinson, Global ducks distribution in 2015 (5 minutes of arc). In V1 ed.; Harvard Dataverse: (2022).

[16] M. Gilbert, G. Cinardi, D. Da Re, W. G. R. Wint, D. Wisser and T. P. Robinson, Global buffaloes distribution in 2015 (5 minutes of arc). In V1 ed.; Harvard Dataverse: (2022).

[17] M. Gilbert, G. Cinardi, D. Da Re, W. G. R. Wint, D. Wisser and T. P. Robinson, Global cattle distribution in 2015 (5 minutes of arc). In V1 ed.; Harvard Dataverse: (2022).

[18] Q.-Q. Zhang, G.-G. Ying, C.-G. Pan, Y.-S. Liu and J.-L. Zhao. Comprehensive Evaluation of Antibiotics Emission and Fate in the River Basins of China: Source Analysis, Multimedia Modeling, and Linkage to Bacterial Resistance. Environmental Science & Technology 49(11) (2015) 6772-6782.

[19] X. Zhou, J. Wang, C. Lu, Q. Liao, F. O. Gudda and W. Ling. Antibiotics in animal manure and manure-based fertilizers: Occurrence and ecological risk assessment. Chemosphere 255 (2020) 127006.

[20] L.-J. Zhou, G.-G. Ying, S. Liu, R.-Q. Zhang, H.-J. Lai, Z.-F. Chen and C.-G. Pan. Excretion masses and environmental occurrence of antibiotics in typical swine and dairy cattle farms in China. Science of The Total Environment 444 (2013) 183-195.

[21] G. Wang, G. Li, J. Chang, Y. Kong, T. Jiang, J. Wang and J. Yuan. Enrichment of antibiotic resistance genes after sheep manure aerobic heap composting. Bioresource Technology 323 (2021) 124620.

[22] Y. X. Li, X. L. Zhang, W. Li, X. F. Lu, B. Liu and J. Wang. The residues and environmental risks of multiple veterinary antibiotics in animal faeces. Environ Monit Assess 185(3) (2013) 2211-2220.

[23] S. Zhi, S. Shen, J. Zhou, G. Ding and K. Zhang. Systematic analysis of occurrence, density and ecological risks of 45 veterinary antibiotics: Focused on family livestock farms in Erhai Lake basin, Yunnan, China. Environmental Pollution 267 (2020) 115539.

[24] Z. Zhu, X. Zhang, H. Dong, S. Wang, S. Reis, Y. Li and B. Gu. Integrated livestock sector nitrogen pollution abatement measures could generate net benefits for human and ecosystem health in China. Nature Food 3(2) (2022) 161-168.

[25] R. E. G. Bohrer, E. Carissimi, D. B. Wolf, O. D. Prestes, R. Zanella, T. M. Rizzetti, D. A. R. Lopez, Ê. L. Machado and D. M. da Silva. Removal of High Concentrations of Veterinary Antibiotics Through Co-composting of Swine Waste. Waste and Biomass Valorization 12(1) (2021) 407-416.

[26] Y.-x. Chu, C.-r. Fang, H. Wang, X.-k. Wu, Y.-j. Gu and J. Shu. Degradation of sulfonamides during anaerobic composting of swine manure. Chemistry and Ecology 33(4) (2017) 339-351.

[27] M. S. Gaballah, J. Guo, H. Sun, D. Aboagye, M. Sobhi, A. Muhmood and R. Dong. A review targeting veterinary antibiotics removal from livestock manure management systems and future outlook. Bioresource Technology 333 (2021) 125069.

[28] Y. Han, L. Yang, X. Chen, Y. Cai, X. Zhang, M. Qian, X. Chen, H. Zhao, M. Sheng, G. Cao and G. Shen. Removal of veterinary antibiotics from swine wastewater using anaerobic and aerobic biodegradation. Science of The Total Environment 709 (2020) 136094.

[29] A. Spielmeyer. Occurrence and fate of antibiotics in manure during manure treatments: A short review. Sustainable Chemistry and Pharmacy 9 (2018) 76-86.

[30] B. Liu, Y. Xie, Z. Li, Y. Liang, W. Zhang, S. Fu, S. Yin, X. Wei, K. Zhang, Z. Wang, Y. Liu, Y. Zhao and Q. Guo. The assessment of soil loss by water erosion in China. International Soil and Water Conservation Research 8(4) (2020) 430-439.

[31] G. P. Weedon, G. Balsamo, N. Bellouin, S. Gomes, M. J. Best and P. Viterbo. The WFDEI meteorological forcing data set: WATCH Forcing Data methodology applied to ERA-Interim reanalysis data. Water Resources Research 50(9) (2014) 7505-7514.

[32] L. Stefan and B. Matthias, ISIMIP3b bias-adjusted atmospheric climate input data (v1.1). In I. Repository, Ed. (2021).

[33] Y. Li, Q. Zhang, J. Baartman, J. van Wijnen, N. Beriot, C. Kroeze, M. Wang, W. Xu, L. Ma, K. Wang, F. Zhang and M. Strokal. The Plastic Age: River Pollution in China from Crop Production and Urbanization. Environmental Science & Technology 57(32) (2023) 12019-12032.

[34] NESSDC, Map of soil texture on a 1-kilometer resolution (2010-2018). In X. Song, F. Liu, G. Zhang and k. Pan, Eds. National Earth System Science Data Center (2016).

[35] M. Pan and L. M. Chu. Adsorption and degradation of five selected antibiotics in agricultural soil. Sci Total Environ 545-546 (2016) 48-56.

[36] S. Thiele-Bruhn. Pharmaceutical antibiotic compounds in soils – a review. Journal of Plant Nutrition and Soil Science 166(2) (2003) 145-167.

[37] S. Hu, Y. Zhang, G. Shen, H. Zhang, Z. Yuan and W. Zhang. Adsorption/desorption behavior and mechanisms of sulfadiazine and sulfamethoxazole in agricultural soil systems. Soil and Tillage Research 186 (2019) 233-241.

[38] J. Tolls. Sorption of Veterinary Pharmaceuticals in Soils:  A Review. Environmental Science & Technology 35(17) (2001) 3397-3406.

[39] M. Cycoń, A. Mrozik and Z. Piotrowska-Seget. Antibiotics in the Soil Environment-Degradation and Their Impact on Microbial Activity and Diversity. Front Microbiol 10 (2019) 338.

[40] H. Dolliver and S. Gupta. Antibiotic losses in leaching and surface runoff from manure-amended agricultural land. J Environ Qual 37(3) (2008) 1227-1237.

[41] L. Wöhler, P. Brouwer, D. C. M. Augustijn, A. Y. Hoekstra, R. J. Hogeboom, B. Irvine, V. Lämmchen, G. Niebaum and M. S. Krol. An integrated modelling approach to derive the grey water footprint of veterinary antibiotics. Environmental Pollution 288 (2021) 117746.

[42] T. Kivits, H. P. Broers, H. Beeltje, M. van Vliet and J. Griffioen. Presence and fate of veterinary antibiotics in age-dated groundwater in areas with intensive livestock farming. Environmental Pollution 241 (2018) 988-998.

[43] S. R. Wegst-Uhrich, D. A. G. Navarro, L. Zimmerman and D. S. Aga. Assessing antibiotic sorption in soil: a literature review and new case studies on sulfonamides and macrolides. Chemistry Central Journal 8(1) (2014) 5.

[44] A. Białk-Bielińska, J. Maszkowska, W. Mrozik, A. Bielawska, M. Kołodziejska, R. Palavinskas, P. Stepnowski and J. Kumirska. Sulfadimethoxine and sulfaguanidine: Their sorption potential on natural soils. Chemosphere 86(10) (2012) 1059-1065.

[45] R. A. Figueroa-Diva, D. Vasudevan and A. A. MacKay. Trends in soil sorption coefficients within common antimicrobial families. Chemosphere 79(8) (2010) 786-793.

[46] L. D. Geohring, D. Gates and S. W. Duiker, Soil and water management: Study Guide., in: D. W. Wolfe and P. A. Ray (Eds.), Cornell University, Northeast Region Certified Crop Adviser (NRCCA), 2016.

[47] MARA, Crop Calendar (in Chinese). In MARA: Ministry of Agriculture and Rural Affairs of the People's Republic of China (2018) Vol. 2022.

[48] L. Wöhler, G. Niebaum, M. Krol and A. Y. Hoekstra. The grey water footprint of human and veterinary pharmaceuticals. Water Research X 7 (2020) 100044.

[49] MOA, Zoning rotational grazing - a new model of scientific grazing (in Chinese). In Ministry of Agriculture and Rural Affairs of the People's Republic of China: Ministry of Agriculture and Rural Affairs of the People's Republic of China (2004) Vol. 2023.

[50] MOA, Feeding management technology for cattle grazing (in Chinese). In China Agricultural Information Network: China Agricultural Information Network (2016) Vol. 2023.

[51] C. Bailey, The overland transport of veterinary antibiotics In Faculty of Civil Engineering, RWTH Aachen, RWTH Aachen: (2015) p 161.

[52] P. Kay, P. A. Blackwell and A. B. A. Boxall. Transport of veterinary antibiotics in overland flow following the application of slurry to arable land. Chemosphere 59(7) (2005) 951-959.

[53] Q. Wang, M. Guo and S. R. Yates. Degradation Kinetics of Manure-Derived Sulfadimethoxine in Amended Soil. Journal of Agricultural and Food Chemistry 54(1) (2006) 157-163.

[54] Y. Zhang, S. Hu, H. Zhang, G. Shen, Z. Yuan and W. Zhang. Degradation kinetics and mechanism of sulfadiazine and sulfamethoxazole in an agricultural soil system with manure application. Sci Total Environ 607-608 (2017) 1348-1356.

[55] N. Wang, X. Guo, J. Xu, L. Hao, D. Kong and S. Gao. Sorption and transport of five sulfonamide antibiotics in agricultural soil and soil-manure systems. J Environ Sci Health B 50(1) (2015) 23-33.

[56] F. H. M. Tang and F. Maggi. Pesticide mixtures in soil: a global outlook. Environmental Research Letters (2021).

[57] B. Bezabih Beyene, J. Li, J. Yuan, Y. Dong, D. Liu, Z. Chen, J. Kim, H. Kang, C. Freeman and W. Ding. Non-native plant invasion can accelerate global climate change by increasing wetland methane and terrestrial nitrous oxide emissions. Global Change Biology 28(18) (2022) 5453-5468.

[58] Y. Zhang and G. S. Marcel, A High-Resolution Global Map of Soil Hydraulic Properties Produced by a Hierarchical Parameterization of a Physically-Based Water Retention Model. In V1 ed.; Z. Yonggen and G. S. Marcel, Eds. Harvard Dataverse: (2018).

[59] W. Jia, W. Qin, Q. Zhang, X. Wang, Y. Ma and Q. Chen. Evaluation of crop residues and manure production and their geographical distribution in China. Journal of Cleaner Production 188 (2018) 954-965.

[60] F. Maggi, F. H. M. Tang, D. la Cecilia and A. McBratney. PEST-CHEMGRIDS, global gridded maps of the top 20 crop-specific pesticide application rates from 2015 to 2025. Scientific Data 6(1) (2019) 170.

[61] M. Strokal, Z. Bai, W. Franssen, N. Hofstra, A. A. Koelmans, F. Ludwig, L. Ma, P. van Puijenbroek, J. E. Spanier, L. C. Vermeulen, M. T. H. van Vliet, J. van Wijnen and C. Kroeze. Urbanization: an increasing source of multiple pollutants to rivers in the 21st century. npj Urban Sustainability 1(1) (2021) 24.

[62] E. Mayorga, S. P. Seitzinger, J. A. Harrison, E. Dumont, A. H. W. Beusen, A. F. Bouwman, B. M. Fekete, C. Kroeze and G. Van Drecht. Global Nutrient Export from WaterSheds 2 (NEWS 2): Model development and implementation. Environmental Modelling & Software 25(7) (2010) 837-853.

[63] M. Strokal, C. Kroeze, M. Wang, Z. Bai and L. Ma. The MARINA model (Model to Assess River Inputs of Nutrients to seAs): Model description and results for China. Science of The Total Environment 562 (2016) 869-888.

[64] X. Meng and H. Wang, China meteorological assimilation datasets for the SWAT model - soil temperature version 1.0 (2009-2013). In T. P. E. D. C. National Tibetan Plateau, Ed. Beijing, China (2018).

[65] Eurostat, Glossary:Livestock unit (LSU). In Eurostat Statistics Explained: Eurostat Statistics Explained (2022) Vol. 2023.

[66] F. Huang, Z. An, M. J. Moran and F. Liu. Recognition of typical antibiotic residues in environmental media related to groundwater in China (2009−2019). Journal of Hazardous Materials 399 (2020) 122813.
